# Structure Diversity and Properties of Some Bola-like Natural Products

**DOI:** 10.3390/md23010003

**Published:** 2024-12-24

**Authors:** Valentin A. Stonik, Tatyana N. Makarieva, Larisa K. Shubina, Alla G. Guzii, Natalia V. Ivanchina

**Affiliations:** G.B. Elyakov Pacific Institute of Bioorganic Chemistry, Far Eastern Branch, Russian Academy of Sciences, Pr. 100-let Vladivostoku 159, 690022 Vladivostok, Russia; makarieva@piboc.dvo.ru (T.N.M.); shubina@piboc.dvo.ru (L.K.S.); gagry@rambler.ru (A.G.G.); ivanchina@piboc.dvo.ru (N.V.I.)

**Keywords:** archaeal bolaamphiphiles, bacterial bola-like products, bolaamphiphiles of marine invertebrates, structures, taxonomic distribution, biosynthesis, properties

## Abstract

In their shapes, molecules of some bipolar metabolites resemble the so-called bola, a hunting weapon of the South American inhabitants, consisting of two heavy balls connected to each other by a long flexible cord. Herein, we discuss the structures and properties of these natural products (bola-like compounds or bolaamphiphiles), containing two polar terminal fragments and a non-polar chain (or chains) between them, from archaea, bacteria, and marine invertebrates. Additional modifications of core compounds of this class, for example, interchain and intrachain cyclization, hydroxylation, methylation, etc., expand the number of known metabolites of this type, providing their great structural variety. Isolation of such complex compounds individually is problematic, since they usually exist as mixtures of regioisomers and stereoisomers, that are very difficult to be separated. The main approaches to the study of their structures combine various methods of HPLC/MS or GC/MS, 2D-NMR experiments and organic synthesis. The recent identification of new enzymes, taking part in their biosynthesis and metabolism, made it possible to understand molecular aspects of their origination and some features of evolution during geological times. The promising properties of these metabolites, such as their ability to self-assemble and stabilize biological or artificial membranes, and biological activities, attract additional attention to them.

## 1. Introduction

**Figure 1:**
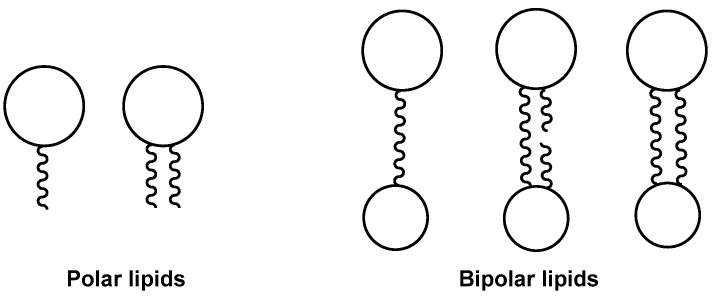
Schematic representation of polar and bipolar natural compounds.

Bipolar natural compounds with polar fragments, located at both ends of the hydrophobic chain (or such chains) connecting them to each other, form a class of natural products known both as structural components of biomembranes and as bioregulators. Schematically, the structures of polar and bipolar lipids are usually shown by pictograms, in which the polar fragments are depicted in circles and the nonpolar “tails” are represented by sinuous lines (Figure 1). Sometimes, some bipolar metabolites with long and flexible chains are called bolalipids, or more generally as “bolaamphiphilic molecules”, deriving from the “bola”—a hunting weapon of the South American residents for throwing at animals. Bolas can wrap themselves around the front legs or neck of animals and help to catch them [1].

In our review, we briefly consider structures and properties of three groups of bola-like natural compounds, namely: (1) bipolar lipids of archaea; (2) corresponding compounds from extremophilic bacteria; and (3) sphingolipid-like and other bipolar metabolites from marine invertebrates. Herein, we also discuss the problems and prospects of further studies and the potential application of such natural compounds and their analogs.

## 2. Bipolar Lipids of Archaea and Some Results of the Studies on Their Structures and Properties

### 2.1. General Information About Archaea and Their Lipids

Considering structures of key biomolecules are the most important taxonomic characters of living systems, in 1977, Woese et al. proposed a three-domain classification of all organisms with the isolation of the domains of archaea, bacteria, and eukarya on the basis of a comparative analysis of membrane constituents, ribosomes, and RNAs of inhabitants of our planet [2,3]. The eukarya includes the kingdoms of plants, animals, and fungi (Plantae, Animalia, and Fungi, respectively); the bacteria have the kingdoms of true bacteria (*Eubacteria*), *cyanobionts* (*Cyanobionta*), and some others; and the archaea domain, according to the later data, consists of the large taxa *Euryarchaeota* (*methanogens*), *Thermoproteota* (formerly *Crenarchaeota*) and *Korarchaeota* (*thermophilic archaea*), *Nitrososphaerota* (formerly *Thaumarchaeota*, chemolithoautotrophic ammonia-oxidizers archaea), *Nanoarchaeota* (can be developed only in co-culture with other archaea), and some others.

Archaea are known as ancient microorganisms that appeared in harsh anaerobic conditions in aqueous hot reservoirs of volcanic origin many hundreds of millions of years ago, when the first unicellular organisms sprang up on our planet [4]. Lipids of their membranes have fundamentally different structures in comparison with similar compounds of bacteria and eukarya. They are constructed from glycerol (or other polyols) and usually fully saturated isoprenoid chains attached to terminal glycerols by ether bonds, rather than from glycerol and fatty acid residues connected by ester bonds, as is typical for bacterial and eukaryotic lipids. Archaeal lipids are characterized by high stability and stereochemical peculiarities because they contain fragments of 2,3-di-*O*-*sn*-substituted glycerol, but not similar residues with 1,2-di-*O*-*sn*-stereochemistry, as in lipids of other organisms. Consequently, substituted glycerol fragments in archaea and in other organisms have antipodal configurations.

Earlier, archaea were grouped with bacteria and collectively called prokaryotes, but after the work of Woese and his colleagues, scientists began to consider them as the third branch of the evolutionary tree of all the organisms, inhabiting the Earth. Several major phenotypes of archaea are known to include halophiles, methanogens, and thermophiles. One more group combines ammonia oxidizers and plays an important role in terrestrial ecosystems, including soils [5]. Archaea are anaerobic microorganisms thriving in the marine environment [6,7,8], which dominate the mesopelagic zone and bottom sediments [9,10]. There are many archaeal taxa living in various thermal springs, lakes, swamps, peatlands, soils, and the gastrointestinal tract of animals [11,12]. At first, archaea were considered extremophilic organisms, since their first representatives were found in hydrotherms. Later, it was shown that they not only inhabit a significant part of the oceanic cold waters and the seabed, withstanding the corresponding environmental conditions, but also are symbionts of various macroorganisms, found in both invertebrates and vertebrates [13,14].

Evolutionary differentiation of lipids (“lipid divide”) into those, containing polyprenyl substituents with ether bonds between them and glycerol, and the metabolites, composed of fatty acid residues linked to glycerol by ester bonds, apparently appeared as a result of the evolution of common ancestors of bacteria and archaea. Obviously, it took place at very early stages of evolution [15]. Molecular fossils, probably characteristic of archaea, were found in sedimentary rocks and microbial mats, for example, branched hydrocarbons and hydroporphinoid nickel-containing coenzyme F430, which participates in the biosynthesis of methane in methanogenic archaea [16,17].

### 2.2. Structure and Taxonomic Distribution of Archaeal Bipolar Lipids with Emphasis on Recent Studies

**Figure 2:**
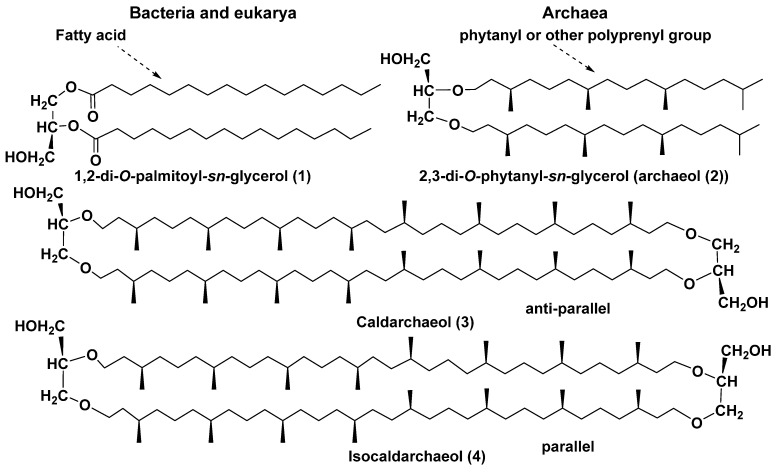
1,2-Di-*O*-palmitoyl-*sn*-glycerol (**1**) of bacteria and eukarya, diether (**2**), and tetraether (**3**, **4**) lipids of archaea.

Unlike common lipids of bacteria and eukarya, for example, 1,2-di-*O*-palmitoyl-*sn*-glycerol (**1**), archaeal lipids usually do not have ester bonds but contain stronger ether bonds. Among the lipids of archaea, di- and tetraether compounds are distinguished; the last mentioned belong to bipolar bola-like natural compounds. Archaeol (**2**), first discovered in *Halobacterium cutirubrum* [18], and caldarchaeol (**3**), first found in *Thermoplasma acidophilum* [19], are examples of diether and tetraether natural compounds (Figure 2). Lipids like **3**, in which glycerol fragments are not connected with phosphate- and/or carbohydrate-containing polar groups, are called core lipids. They are known as biosynthetic precursors of more polar intact membrane lipids and are formed from them during cellular lysis.

Structural types of the currently known bola-like core lipids of archaea are numerous and very diverse: they differ from each other in the length of isoprenoid chains, presence or absence of double bonds, as well as of five-membered and six-membered cycles in their chains. Mainly C_20_–C_20_ (C_40_) isoprenoid moieties of these natural products are formed as a result of dimerization (coupling) of geranylgeranyl residues in the “head-to-head” manner.

Caldarchaeol and related lipids have 72-membered macrocycles, formed of two octaprenyl chains and glycerol residues. As was shown later [20], caldarchaeol is actually a mixture of two regioisomers, difficult to separate: caldarchaeol proper (**3**) and isocaldarchaeol (**4**), both belonging to the class of natural products known as glycerol dialkyl glycerol tetraethers (GDGTs). Although isomers **3** and **4** are practically inseparable, they were identified in many species by degradation methods, e.g., conversion of caldarchaeol and isocaldarchaeol ditosylates into the corresponding polyisoprene iodides with the cleavage of ether bonds at the treatment with HI, followed by reaction with potassium butylate in dimethyl sulfoxide. Another approach to their structure identification consists of the cleavage of ether bonds by BCl_3_ in methylene chloride. In both cases, the subsequent identification of the resulting products was carried out using HPLC/MS and NMR spectroscopy [20]. The stability of these metabolites and their ability to function as the major membrane components, forming a monolayer spanning the lipid phase, explain their widespread distribution in extremophilic and other groups of archaea, which emerged as a result of the evolution of extremophiles and populated not only hydrotherms, but also cold ocean waters, sediments, soils, and other habitats.

Since the 1980s, results of the studies on isolation, structure determination, and properties of bipolar tetraether metabolites of archaea were repeatedly discussed in review articles [21,22,23,24,25,26], albeit new representatives of this class, whose structures were not mentioned in these articles, also attracted attention. At the same time, a complete solution of structural problems, especially those concerning the stereochemistry of such compounds, is possible only after the isolation of individual target metabolites and/or using their syntheses and 2D NMR spectroscopy and cannot be achieved only by chromatographic and mass spectrometry methods. That is why the number of natural compounds of this class with fully established chemical structures remains small. On the other hand, the structural diversity of archaeal bipolar metabolites increases every year, mainly as a result of their indication by HPLC/MS studies. However, some important particularities, such as the stereochemistry of found natural products, often remain unknown.

A key stage in the biosynthesis of tetraether lipids from diether compounds is the dimerization of archaeol-type lipids under the action of tetraether lipid synthase (TES) and closely related enzymes, recently discovered by Zeng et al. TES is an S-adenosylmethionine enzyme (SAM-enzyme), which catalyzes the formation of the C–C bond between terminal sp^3^-hybridized carbon atoms of the lipid tails of two diether molecules via the radical mechanism, involving the participation of iron sulfides, to form bipolar lipids. At the same time, attempts to obtain a mutant form of the archaeon *Sulfolobus acidocaldarius* by deleting the gene-encoding TES were unsuccessful, indicating that enzymes of this type could be essential for the survival of their producers. Expression of TES homologues in other microorganisms led to the biosynthesis of GDGTs, such as **3** and **4**, in the methanogenic archaeon *Methanococcus maripaludis*, which accumulates archaeol (**2**), a precursor of tetraether lipids. Bioinformatic analysis revealed that TES homologues are encoded in archaea and in some bacteria, producing in the latter case so-called branched GDGTs (brGDGTs) [27].

Simultaneously, Lloyd et al. reported that the gene product of mj0619 from the archaeon *Methanocaldococcus jannaschii*, designated GDGT–macrocyclic archaeol synthase (GDGT-MAS), proved to be also a radical SAM-enzyme, responsible for biphytanyl chains biosynthesis in GDGT lipids.

The presence of four metallocofactors, including three [Fe_4_S_4_] clusters and one mononuclear rubredoxin-like iron ion, was established in this enzyme. Moreover, the structure of GDGT-MAS, obtained by heterologous expression in *E. coli*, was determined by X-ray crystallography and mass spectrometry. The molecular mechanism of GDGT-MAS action enables the reductive cleavage of SAM to give methionine and a 5′-deoxyadenosyl 5′-radical. The latter initiates the formation of C–C bonds between two terminal carbon atoms in biphytanyl chains by the detachment of hydrogen atoms [28].

Structures **3** and **4** were confirmed by their chemical syntheses, including those carried out in 1998 by Eguchi et al. [29], who obtained both these bipolar compounds in 45 stages each [29]. Recently, Andringa et al. [30] reported partial syntheses of caldarchaeol and isocaldarchaeol using as an initial substance 35 g of all-*E*-geranylgeraniol, obtained by heptane extraction of 10 kg annatto (*Bixa orellana*) seeds, followed by column chromatography. In these syntheses, asymmetric hydrogenation using chiral ruthenium [31] and iridium catalysts [32] to create the corresponding asymmetric centers were used. As a result, isocaldarchaeol (**4**) was obtained in 20 steps with a yield of 30%. Unsymmetrical caldarchaeol (**3**) was synthesized in 28 steps, which was 17 steps less than in the previously reported total synthesis by Eguchi et al. The realization of these synthetic pathways opened the possibility for accumulation of **3** and **4** for their subsequent use in scientific research and/or in medicine and biotechnology.

**Figure 3:**
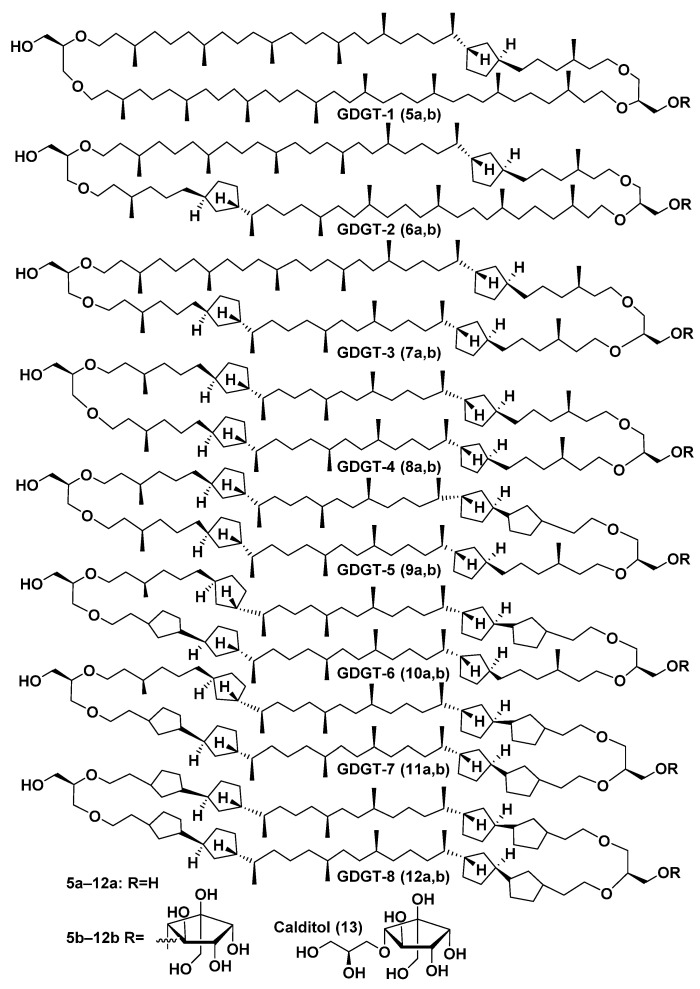
GDGTs **5a**–**12a** and glycerol-calditol bipolar lipids **5b**–**12b** and calditol (**13**).

Analogs of caldarchaeol with cyclic, mainly cyclopentane fragments in their chains, such as **5a**–**12a** (Figure 3), were found in many thermophilic, methanogenic, and ammonia-oxidizing archaea, as well as in bottom sediments. For example, they were indicated in the archaeon *Cenarchaeum symbiosum*, a symbiont of the sponge *Axinella mexicana*, extracts of which along with other biological sources were used to isolate these bolalipids [33].

Experiments with isotopically labeled precursors demonstrated that benthic archaea are capable of biosynthesizing de novo only glycerol fragments of GDGTs, while isoprenoid chains may be obtained by them in the ready forms from the bottom sediments themselves, where the content of such compounds is sufficiently high, and they are used for the biosynthesis [34].

Cyclopentane-containing bipolar compounds are formed as a result of intrachain cyclizations in **3** and **4** or related compounds. Each of them is a mixture of two regioisomers, like **3** and **4** themselves, but these products of cyclization, as a rule, were not isolated individually and studied mainly by HPLC/MS [35,36].

Bipolar bolalipids (**5b**–**12b**), belonging to another series of core bipolar lipids, contain the fragment of a polyol (**13**), first defined as *n*-nonitol and later redesignated as calditol, instead of one of the glycerol residues (Figure 3). Compound **13** was found in the composition of such metabolites of archaea of the order Sulfolobales, and its structure was clarified by synthesis. Calditol includes a pentacyclic fragment linked to glycerol by an ether bond, which is not cleaved by BF_3_ but can be reduced by HI. Of the 4 of its diastereoisomeric variants synthesized, only 1 was completely identical to this natural product found in *Sulfolobus solfataricus*, which allowed for establishing the structure of **13** [37]. The clarification of positions of five-membered rings in the above-mentioned bipolar lipids was performed by HI-LiAlH4 degradation. As a result of this reaction, ether bonds in initial bolalipids were cleaved, and MS fragmentation of the obtained isoprenoid products was used for their structure analyses.

The number of five-membered rings in the lipids of thermophilic archaea depends on the temperature in habitats and varies from 1 to 8. Their presence compacts the corresponding membranes and makes them more stable. The more pentacyclic fragments are present in the hydrophobic chains of the corresponding lipids, the higher the temperature at which the membranes retain the liquid crystalline state and the barrier properties, necessary for the survival of their producers [38]. Most likely, calditol-containing lipids, such as **5b**–**12b,** are required for the acid tolerance of their producers [39].

Two GDGT ring synthases, GrsA and GrsB, were identified in the above-mentioned archaea, and both enzymes were proved to be SAM proteins catalyzing cyclization occurring via a free radical molecular mechanism. GrsA catalyzes the formation of a five-membered ring at position 7, whereas GrsB introduces such a cycle at the position 3 of parent core lipids. Phylogenetic analysis of the corresponding genes showed that the dominant sources of **5a,b**–**12a,b** in oceanic waters belong to the wide-spread marine archaea Thaumarchaeota rather than to Euryarchaeota, which explains the accumulation of these compounds in bottom sediments [40].

**Figure 4:**
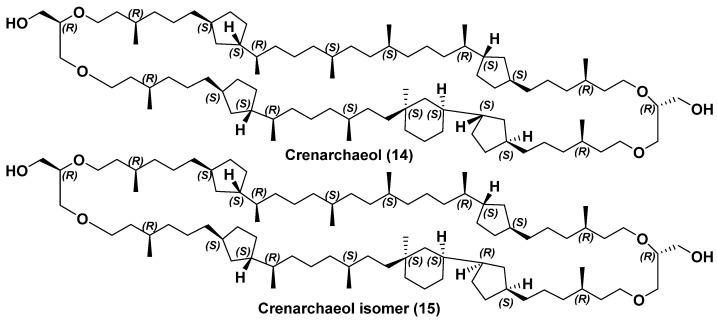
Crenarchaeol (**14**) and crenarchaeol isomer (**15**); both structures are shown for parallel conformations.

Bipolar metabolites with cyclohexane rings, produced by ammonia-oxidizing archaea, were also found. The first compound of this type with one six-membered and four five-membered rings was named crenarchaeol (**14**) (Figure 4) [33]. Crenarchaeol contains a 66-membered macrocycle and 12 asymmetric centers in it. This compound was found in the mixture with **3**, **5a**, and **7a** in a sample of Arabian Sea bottom sediment, collected from a depth of about 1000 m off the coast of Yemen as well as in lipids of the hyperthermophilic archaeon *Sulfolobus solfalaricus*. Moreover, the same bipolar metabolite was identified by the HPLC/MS method in an archaeal symbiont of the sponge *Axinella mexicana*, treated by boiling in 2N HCl. Its structure was supported using 1D and 2D NMR spectra and HPLC-APCI/MS data (а high-performance liquid chromatography–atmospheric pressure chemical ionization–mass spectrometry). Configurations of asymmetric centers were proposed mainly on the basis of NMR studies by comparing the chemical shifts and multiplicity of proton signals with those in the spectra of related bipolar lipids. In order to ultimately confirm the structure and stereochemistry of **14**, its total synthesis was carried out [41], but unfortunately, the NMR spectra of the synthesized product and natural **14** differed from each other. As a result of the examination of the NMR spectra of the synthesized product itself and calculated spectra of its stereoisomers with other configurations in a six-membered cycle, the configuration of the quaternary asymmetric center embedded in cyclohexane ring was revised and proposed to be of S.

It is believed that crenarchaeol is one of the predominated bipolar lipids in the Earth’s biosphere, common among oceanic non-thermophilic archaea. Many bolalipids of this type were found in archaea capable of living in non-extreme conditions. Pelagic archaea, for example, those producing **14** and its analogs, are probably one of the most widespread groups of microbes (totally, 10^28^ cells). This number is of the same order as the number of all bacteria in the World Ocean [9].

Moreover, a new isomer (**15**) of crenarchaeol [42], which has not a *trans*-configuration, but a *cis*-configuration in a five-membered ring, located nearby to the cyclohexane fragment, was isolated from the marine surface sediment. Its structure, shown above, was proposed by the application of ^13^C NMR data; it was revised by us in this review in accordance with [41]. The history of determining the structures of **14** and **15** is very instructive and shows that not only MS data, but even NMR data, are sometimes insufficient to establish all structure peculiarities of this type complicated for analysis of bola-like natural products, especially if they were isolated from the corresponding natural sources as mixtures of stereo- or regioisomers (Figure 4).

A careful examination of the distribution of isoprenoid GDGTs (isoGDGTs) in different biological sources and their application as molecular tools for finding out the characteristic patterns of microbial evolution, as well as indicators of various environmental parameters, such as pH and temperature, has been reviewed several times in recent years (see, e.g., [43,44]). The determination of the content of tetraether compounds with different numbers of cyclopentane and cyclohexane fragments in their fractions made it possible to analyze changes in the temperature of oceanic surface waters and bottom sediments, especially over the last century in connection with global climate changes. The advantages of this method of thermometry, based on the analysis of archaeal bipolar lipid compositions, are the wide distribution of these microorganisms in various ecological niches and the resistance of the corresponding bola-like metabolites to decomposition.

The first formula for calculating the paleotemperature tetraether index (TEX_86_) (most of these compounds have 86 carbon atoms) was proposed by Schouten et al. in 2002 [45]. It was based on the relative content of certain bipolar lipids in sediments and after that was refined several times [46,47,48].

(1) $${\mathrm{TEX}}_{86}=\frac{\left[GDGT-2\right]+\left[GDGT-3\right]+[Cren^{\prime }]}{\left[GDGT-1\right]+\left[GDGT-2\right]+\left[GDGT-3\right]+[Cren^{\prime }]}$$

Abbreviation GDGT-n represents isoprenoid GDGTs with n number of cyclopentane rings, renarchaeol and its isomer are abbreviated as Cren′.

**Figure 5:**
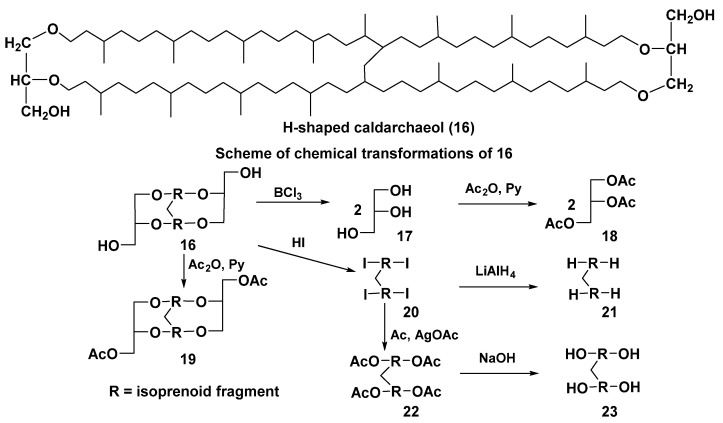
The structure of H-shaped caldarchaeol (**16**) and its chemical transformations.

In addition to intrachain cyclization, leading to the formation of five- and six-membered rings, interchain conversions also take place in the processes of biosynthesis of bipolar compounds. Actually, the so-called H-shaped caldarchaeol (**16**) (Figure 5) from the hyperthermophilic methanogenic archaeon *Methanothermus fervidus* [49] is obviously a result of the interaction between a methyl group in the middle of one isoprenoid moiety and a double bond in another chain. Indeed, it has a molecular weight of 2 units less than caldarchaeol (**3**), and the ^1^H NMR spectrum of 16 lacks a three-proton signal of one methyl group and contains signals of 15 methyl groups instead of 16 compared to that of 3.

While establishing its hypothetical structure, chemical transformations, shown in Figure 5, were applied. For example, cleavage of ether bonds under the action of BCl_3_ led to the formation of glycerol (**17**) as one of the main water-soluble products, after acetylation identified by GC as triacetate (**18**). The treatment of **16** with acetic anhydride in pyridine gave diacetate (1**9**) and with HI acid yielded tetraiodide (**20**). The latter was converted by LiAlH_4_ reduction into hydrocarbon (**21**). The product **20** was also converted into tetraacetate (**22**) with silver acetate in acetic acid and then into tetraol (**23**) by saponification of **22** with alkali. Unfortunately, it failed to determine the exact position of a single-carbon bridge in **16** and the stereochemistry of its stereogenic centers.

**Figure 6:**
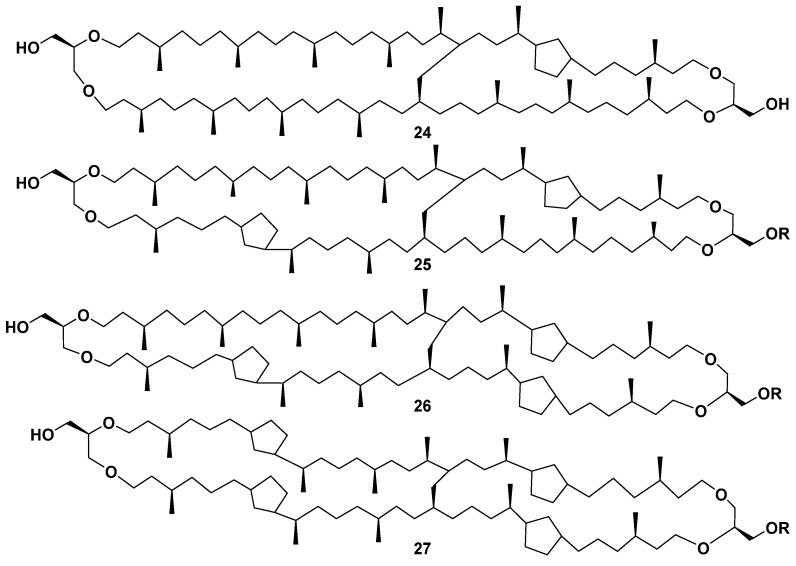
The structures of some other H-shaped bipolar lipids **24**–**27**.

Compounds **24**–**27**, found in cultured thermoacidophilic archaeon *Candidatus* “*Aciduliprofundum boonei*” from deep-sea hydrothermal vents, proved to be cyclopentane-containing lipids belonging to the same structural series (Figure 6) [50].

Representatives of a new group of archaeal bola-like natural products from estuarine and deep-sea bottom sediments were discovered by Zhu et al., who identified their unprecedented backbones with additional methyl or ethyl groups introduced into a glycerol residue using preparative HPLC/MS. The corresponding bipolar lipids were named butanetriol and pentanetriol dialkyl glycerol tetraethers (BDGTs and PDGTs) [51].

**Figure 7:**
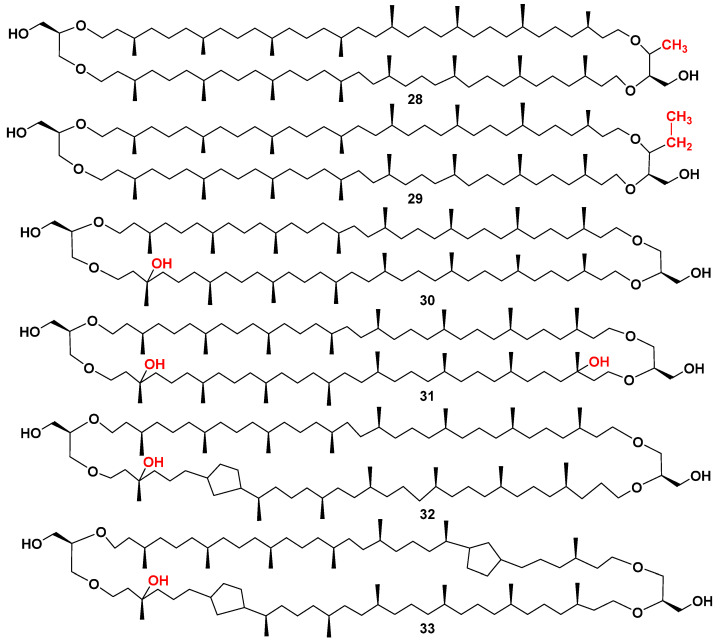
The structures of butanetriol (**28**), pentanetriol (**29**), and some hydroxylated (**30**–**33**) archaeal core lipids.

Sometime later, the corresponding bipolar metabolites containing only one terminal glycerol residue and another terminus, represented by butanetriol or pentanetriol fragments, as in **28** and **29** (Figure 7), were detected in extracts of *Methanomassiliicoccus luminyensis,* only cultured archaeal methanogenic species of the order Methanomassilicoccales. Their structural analysis was facilitated by HPLC/MS [51]. The appearance of 1,2,3-butanetriol or 1,2,3-pentanetriol fragments in archaeal bipolar lipids was suggested to be adaptive traits of some methanogenic archaea to energy-limited environments [52].

Studies on such bipolar lipids, additionally, alkylated in glycerol residue and isolated from the cultured *M. luminyensis,* showed that an additional methyl group is introduced to C-3 of a glycerol residue during the formation of the butanetriol fragment. Content of the butanetriol- and, particularly, pentanetriol-containing tetraethers, such as **28** and **29**, was increased in the stationary growth phase of the culture under conditions of nutrient and energy deficiency. The C-3 position of methylation in a modified glycerol residue of these lipids was unambiguously confirmed using the s2D NMR technique with high-field NMR experiments. Moreover, marine sediments, collected in the Mediterranean Sea and Black Sea during marine expeditions, were examined for isotopic compositions and abundance of their BDGTs and PDGTs in both intact polar and core lipids of sediments, confirming the wide geographical distribution of such archaeal metabolites in bottom sediments [53].

The examination of the biosynthesis of BDGTs and PDGTs, main membrane lipids of the methanogenic archaeon *M. luminyensis*, was carried out using stable isotope labeled methyl-^13^C-methionine. These experiments showed that biosynthesis proceeds through a radical mechanism by the transfers of terminal methyl groups of methionine into common archaeal membrane GDGTs with the probable participation of a SAM enzyme [54].

Mono- and dihydroxylated GDGT derivatives of bipolar lipids, constituting another important series of bola-like natural products, are also widely represented in core and intact bipolar constituents of marine sediments. Two-dimensional^1^H-^13^C NMR spectra on small quantities of analytes, isolated from marine sediments, helped to identify the major compound (**30**) with a tertiary hydroxyl group as a monohydroxy-GDGT (OH-GDGT-0) [55]. The content of **30**–**33** (Figure 7) and related compounds and their compositions in bottom sediments and lipidomes of planktonic Thaumarchaeota (totally, with 118 different lipids) [56] may be used to reconstruct long-term temperature changes in surface layers of sea waters. New calibrations for more accurate reconstruction of paleotemperatures also used information concerning hydoxylated bipolar lipids [57].

### 2.3. Intact Membrane Bipolar Lipids from Archaea

Unlike core lipids, water-soluble intact membrane lipids contain polar phosphate- and/or carbohydrate-containing substituents linked to terminal fragments, and, as a result, these compounds are in majority asymmetric. Being more labile and represented by more complicated mixtures, they are difficult to isolate in individual states. It proved to be that, in many cases, their phosphate-containing polar termini were similar to those of phospholipids from bacteria and eukaryotes. Myo-inositol phosphate, choline, ethanolamine, serine, additional glycerol, and phosphate groups were found in their polar moieties. Usually carbohydrate-containing fragments consist of one to four monosaccharide residues, such as α- and β-D-glucopyranose, α- and β-D-galactopyranose, β-D-galactofuranose, α-D-mannopyranose, and others. The sugars in these fragments can be sulfated, for instance, as in 3-sulfate of D-galactopyranose, as well as in 2- and 6-sulfates of D-mannopyranose.

**Figure 8:**
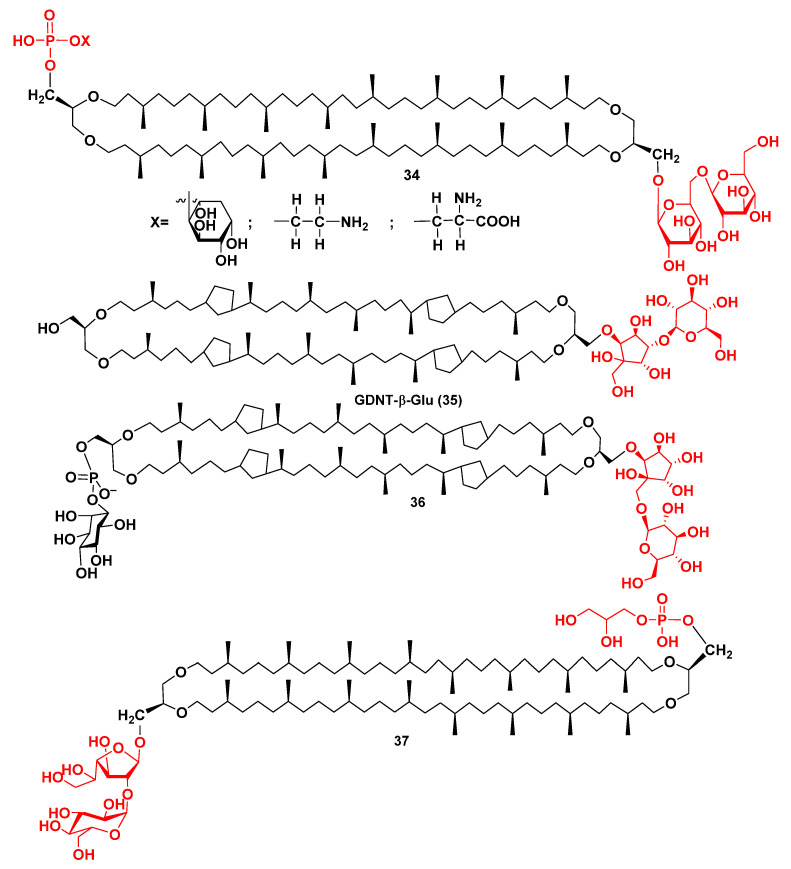
Some intact lipids from *Methanobacterium thermoautotrophicum* (**34**), from the *Sulfolobus* genus (**35**, **36**), and from *Pyrococcus furiosus* (**37**).

The significant structural diversity of such compounds and the dependence of their structures upon the taxonomic position of producers were revealed mainly in the early stages of studying the archaeal membrane bipolar lipids. Structural analysis of mixtures consisting of such compounds was usually carried out by HPLC/MS and degradation methods. Figure 8 shows the general formula **34** of a group of such lipids from *Methanobacterium thermoautotrophicum* [58] as an example of this type of intact membrane constituent.

Until now, there were limited data concerning the exact structures of individual archaeal intact bipolar lipids, determined by NMR spectroscopy and/or confirmed by organic synthesis. Recently, the major membrane lipid of thermophilic archaea, belonging to the genus *Sulfolobus,* was shown to be a tetracyclopentane derivative, so-called GDNT-β-Glu (**35**), containing a glucopyranose residue attached to the calditol (Figure 8). Its structure was determined by Scholte et al. after the isolation of **35** and analyses of its NMR and MS spectra [59]. They studied membrane lipids from *S. solfataricus*, *S. acidocaldarius*, and *S. metallicus*, cultured at 85 °C, 70 °C, and 75–80 °C, respectively. In each of these cases, mixtures of related compounds, having from 4 to 5–7 cyclopentane rings were obtained. At that, 65% of the derivative **35** in a fraction of intact lipid, containing 4 cyclopentane fragments, was found from *S. metallicus*. This fraction was chosen for purification and further structural study on this compound.

Structure elucidation of **35** was difficult due to the poor solubility of this intact lipid and the coincidence of signals of the calditol and the carbohydrate fragment with the signals of solvent (deuterated THF). The exact position of glucose attached to calditol was established using HMBC experiments. NMR spectra of **35** unexpectedly showed that the bond between β-glucopyranose and calditol is connected rather with CH than the CH_2_ group of the pentacyclic fragment in calditol, in contrast with commonly considered structure **36** for similar compounds [59].

However, the majority of the studies on this type of compound as well as structural analyses of archaeal lipidomes were carried out without isolation of individual bipolar lipids using various combinations of modern chromatographic methods and the subsequent analysis of the indicated substances by mass spectrometry with ESI, APCI, or MALDI ionizations. Over the past two decades, four major platforms, based on the application of mass spectrometry to bipolar lipids and related natural products, have been used: GC/MS, UHPLC/MS, MALDI/MS, and shotgun lipidomics. In these studies, principal difficulties and problems were associated with complications in sample preparation, exact identification of the structure of co-eluted isomeric and isobaric lipids, and elucidation of structures of earlier unknown metabolites [60,61].

The corresponding investigation provided new data on lipids from archaea, for instance, during structure determination of an intact polar caldarchaeol derivative (**37**) (Figure 8), containing phosphoglycerol and dihexose fragments at termini of the molecule, which was identified along with a great variety of other lipids in the marine hyperthermophilic archaeon *Pyrococcus furiosus*, whose optimal growth temperature was of 100 °C [62].

Similar studies were carried out also on two species belonging to the same genus, namely *P. furiosus a*nd *P. yayanosii*, showing a great structural diversity of their lipidomes as well as the presence of large sets of bipolar caldarchaeol derivatives (totally, 19 membrane lipids of this type were found) [63]. The most of archaeal intact bipolar metabolites of these microbes contain a carbohydrate moiety with β-D-glucopyranose and/or β-D-galactopyranosyl-β-D-glucopyranose as head groups and free or phosphate-substituted opposite *terminus*.

Almost all the studied archaea were shown to be able to biosynthesize both tetraether and diether lipids; meanwhile, the corresponding monolayer and bilayer membrane fragments in them were laterally segregated as, for example, in the hyperthermophilic archaeon *Thermococcus barophilus*. This type of membrane organization is probably an ancestral feature of archaea. It is likely that each such domain may dock a particular membrane protein [64].

*T. barophilus* is a representative of hyperthermophilic piezophiles, growing at temperatures up to 103 °C and pressures up to 80 MPa. HPLC/MS revealed caldarchaeol and archaeol as major core lipids of this species. The increasing pressure and decreasing temperature in its culture led to a growth of concentration of archaeol derivatives in the corresponding lipidome [65].

### 2.4. Taxonomic Distribution of Bola-like Metabolites in Archaea, Some of Their Properties and Application, Archaeosomes

Bipolar membrane lipids were found in most of the archaeal taxa, with the exception of representatives of the order Halobacteriales belonging to the class Haloarchaea. The most abundant tetraether caldarchaeol (**3**) are widespread in methanogenic archaea, while bipolar lipids with 1–4 cyclopentane rings were reported from a number of species of thermophylic Crenarchaeota and Thaumarchaeota [66,67]. Unusual ‘‘H-shaped’’ glycerol monoalkyl glycerol tetraethers (GMGTs) with several or no cyclopentane fragments seem to be characteristic of hyperthermophilic Euryarchaeota, Crenarchaeota, but generally, the content of such compounds in their extracts is not so high. The relative abundance of bipolar lipids in peat and soil positively correlates with temperature as well as with anaerobic conditions of their inhabiting [68].

Evolution, which resulted in a diversity of archaeal bipolar membrane lipids, allowed these microorganisms to adapt to the environmental conditions in which they survived. The ethereal nature of the chemical bonds between glycerol and saturated isoprenoid chains made archaeal membrane lipids stable over a wide pH range. The unusual stereochemistry in glycerol fragments led to the resistance of these bipolar compounds against the action of phospholipases. Numerous methyl branches prevented the transition of archaeal lipids from the liquid crystalline to the crystalline state.

Chains of bipolar lipids, spanning the membranes of their producers, are organized predominantly into single monolayer in contrast to the bilayer organization of biomembranes in bacteria and eukarya. This makes membranes, containing such bola-like compounds stronger and tougher in extreme environmental conditions. Unusual properties of archaeal bipolar lipids have attracted much attention from scientists interested in many intriguing questions, such as the origin of life on Earth, main features and peculiarities of molecular evolution, long-term climatic changes, and the possibility of practical application of these metabolites themselves as well as their synthetic analogs.

Theoretical calculations, based on molecular dynamics methods and other data showed that bipolar natural products, such as bolalipids, can form structures with U- and O-shaped conformations in archaeal membranes. Their presence allows these membranes to maintain a liquid crystalline state with high thermoplasticity in a wide temperature range from 0 to 100 °C, to be tightly packed, extremely durable, and possess very low water and ion permeability. These natural products and their synthetic analogs are promising for application in biotechnology and material science, as well as for the design of new and unique membrane nanosystems [69]. Model systems, such as vesicular and planar membranes, made from total or partly purified archaeal bipolar lipids, as well as from similar synthetic compounds, are frequently used in studies on properties and opened new possibilities for their application in medicine and biotechnology.

The thermoacidophilic genus *Sulfolobus* has a unique composition of bipolar lipids suitable for the production of archaeosomes (liposomes, constructed from archaeal lipids) with promising medicinal and biotechnological potentials due to chemical and physical stability towards high temperature and acidic pH. Temperature stability is an important advantage in the processing and storage of archaeosomes, whereas acid resistance prevents their degradation in the stomach at oral application [70]. Unilamellar and multilamellar self-assembled archaeosomes, prepared from the archaeal lipids, can form spherical vesicles, known as one of the variants of vesicular delivery systems [71]. The micro-review of Jacquemet et al. described the potential of application of archaeal tetraether lipids not only in biotechnology but also for drug/gene delivery, vaccines, preparation of ultrathin layers and biosensor design, for stabilization of various delivery systems against oxidative stress, high temperature, alkaline pH, action of phospholipases, bile salts and serum environments [72]. Archaeosomes are non-toxic and more stable compared to liposomes, constructed from ordinary ester phospholipids. Moreover, the addition of archaeal or similar synthetic compounds to conventional lipids to impart stability to liposomes made it possible to obtain more stable and affordable hybrid archaeosomes. In general, archaeosomes can be considered a new generation of delivery systems with improved properties. These analogs of more known liposomes can be prepared by traditional methods developed for the production of liposomes via mechanical dispersion, lipid hydration, sonication of lipid films, extrusion, reverse phase evaporation, allowing the encapsulating of various chemicals, for example, antigens. They form a suitable matrix for the function of various exogenic substances, including enzymes and low molecular weight compounds. A strong response of CD4+ and CD8+ cytotoxic lymphocytes to entrapped antigens with the application of suitable archaeosomes, composed of bipolar lipids like caldarchaeols, was shown to continue for 50 weeks. Several businesses were interested and invested in the practical use of archaeosome-based products against various diseases [73,74].

Recently, new promising data regarding the application of natural bola-like archaeal tetraethers for the delivery of various drugs, antioxidants, genes, vaccines, proteins, and peptides have been reviewed by Santhosh and Genova [75] and Chong et al. [76].

Both conventional and hybrid archaeosomes, consisting of either tetraether lipids or tetraethers and conventional lipids, are self-organizing molecular systems. They target cells of the phagocytic system and, being weakly immunogenic, can nevertheless be used as adjuvants and for delivery of various antigens to stimulate the immune system. In particular, they can usually be considered promising tools for the development of new vaccines [77]. An interesting finding regarding the potential application of archaeal bipolar natural products was recently reported by Ayesa and Chong [78]. They constructed stable, but thermosensitive hybrid archaeosomes from the polar lipid fraction E (PLFE) of *S. acidocaldarius* and synthetic dipalmitoylphosphatidylcholine (DPPC) in the ratio of 3:7 with the included anticancer drug doxorubicin (DXO). An abrupt release of DXO, entering into tumor cells, was observed followed by a considerable increase in cytotoxic action when MCF-7 breast cancer cells were treated with these hybrid archaeosomes. Authors suggested that DPPC domains melted and PLFE domains flip-flopped, when the temperature was raised from 37 to 42 °C. Thus, a potentially useful approach was proposed for the treatment of tumor cells at the temperature range, that earlier was clinically approved for mild hyperthermia of tumors [78].

## 3. Natural Bola-Amphiphilic Compounds of Some Anaerobic Bacteria, Peat and Soil

Аnaerobic bacteria are widespread and include thermophilic, psychrophilic, acidophilic, alkaliphilic, halophilic, barophilic, and other species. These microbes were indicated frequently in fresh-water habitats, such as hot springs, peat bogs, lacustrine sediments, soils, etc., and proved to produce unusual bola-like metabolites, providing survival of their producers in environmental conditions [79]. It should not be completely unexpected if similar compounds will be found on other planets, where living microorganisms may exist.

Probably, the origin of extremophilic microorganisms was associated with the emergence of two domains of life (bacteria and archaea) from the so-called Last Universal Common Ancestors (LUCAs), ancient primitive microorganisms that inhabited the primeval biosphere and arose as a result of abiotic synthesis. It was suggested that the evolution of LUCAs led to the appearance of various types of membrane lipids in them, followed by division into groups of precursors of archaea and bacteria with different membrane components [15]. In contrast with structures of the conventional lipids of most bacteria and eukarya, as well as with those of bipolar lipids of archaea, principally other hydrocarbon chains, constructed of predominantly long polybranched fatty acids (brFAs) with ether instead of ester bonds, turned out to be the characteristic structural features of membrane constituents of some anaerobic bacteria, which provided them good barrier properties and a liquid crystalline state of their membranes [79]. Membranes in such bacteria are composed of ether- and/or sometimes ester-linked residues of branched fatty acids attached to glycerol-3-phosphate, contrary to archaeal membranes, consisting of isoprenoid chains, ether-linked to glycerol-1-phosphate [80].

Bipolar bola-like structures of natural products from some bacteria could be formed by one of two biochemical pathways: (i) either by dimerization of their hydrophobic chains by tail-to-tail under the action of special enzymes, like that in archaea, or (ii) via omega-oxidation of suitable fatty acids [81]. In both cases, these transformations are accompanied by other conversions. Thus, in contrast with archaea, the branching of hydrocarbon chains in extremophilic bacteria was achieved as a result of the non-isoprenoid biosynthesis through the evolutionary selection and further conversions of suitable brFAs.

**Figure 9:**
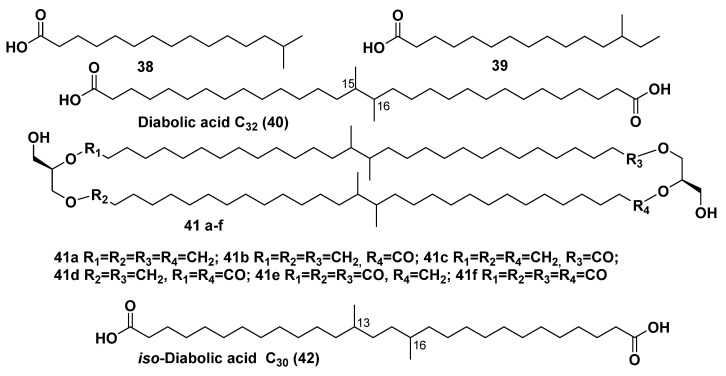
Some branched FAs (**38**, **39**), diabolic acid (**40**), brGDGTs based on diabolic acid (**41a**–**f**), and *iso-*diabolic acid (**42**).

The simplest way to ensure the branched chains of natural products in bacterial membranes is to use common brFAs for their biosynthesis, for instance, those with *iso*- and *anteiso*-structures, such as **38** and **39** (Figure 9). It should be noted that the residues of *iso*- and *anteiso*-FAs were also found in lipids of different microbial and/or non-microbial producers [82].

Another group of branched FAs incorporates the long-chain branched dicarboxylic acids, which were found as constituents of membrane metabolites of hyperthermophilic members belonging to the order Thermotogales, one of the early lineages in the domain of bacteria. Diabolic acid (**40**) and as well as its homologs and derivatives are long-chain acids, containing demethylated fragments with two adjacent methyl groups in the central parts of their chains, which combine into this group. Structurally, they were identified as an intrinsic part of phospholipids isolated from the bacteria *Butyrivibrio* spp. [83]. The type of bipolar lipids, based on **40** and related compounds, was identified by Sinninghe Damste’s group using HPLC/MS in bacteria belonging to the order Thermotogales with an upper growth limit of about 90 °C. Core lipids with diabolic acid residues in their hydrophobic chains were identified in the membranes of nine species of this ancient lineage of bacteria. Since Thermotogales species are assumed to have arisen early during the evolution of life on Earth (that was based on consideration of their position on the phylogenetic tree of life), obtained data on their FAs compositions suggested that the ability to produce both ether- and ester-containing bipolar lipids (general formula **41**) also appeared in the early stages of microbial evolution [84].

Changes in core and intact lipids obtained at culturing *Termotoga maritima* at different temperatures were examined and physiological conditions of the biosynthesis of their membrane-spanning lipids were clarified. Both the growth phase of the bacterium and temperature of culturing influenced the biosynthesis of tetraesters, tetraethers, and mixed ether/ester membrane-spanning core lipids (general formula **41**) as well as of the corresponding intact lipids with phosphoglycerol head group, derived from diabolic acids [85].

*iso*-Diabolic acid (**42**) (Figure 9) and related membrane-spanning lipids are known as members of a subgroup of bacterial long-chain branched bipolar natural products. Natural compounds of this type were identified in some bacteria belonging to the phylum Acidobacteria, one of the most diverse taxa in the domain of bacteria. The acid **42** was not extractable from the corresponding bacterial cells, but along with its derivatives was released after both acid and alkaline hydrolyses. Compound **42** was found in substantial amounts in 3 of 17 strains, studied by GC/MS and HPLC/MS analyses [86].

Bacterial brGDGTs (**43**–**51**), biosynthesized from **42**, have the 1,2-di-*O*-alkyl-*sn*-glycerol-configuration on the contrary with archaeal bipolar lipids. These compounds (Table 1), identified in mesophilic bacteria, thriving in peat, have characteristics of bola-lipids structural peculiarities such as bipolarity and long flexible chains. The presence of cyclopentane fragments, clearly demonstrated by NMR data, makes them look like corresponding archaeal metabolites [87]. The abundance of brGDGTs in the anoxic parts of peat bogs supports their origin from anaerobic bacteria, mostly belonging to the above-mentioned phylum Acidobacteria, which proved to be the dominant group of bacteria, found in peat [88].

Additional inclusion of methyl groups into hydrophobic chains in the processes of their biosynthesis complicates the corresponding mixtures and leads to problems in the analysis of their structures due to difficulties in the isolation of these lipids. Therefore, their structure identification requires the application of modern methods of separation and molecular analysis along with chemical transformations. For example, a brGDGTs fraction from a Siberian peat sample was separated by HPLC and, after that, the obtained substances were subjected to reductive cleavage of ether bonds (57% HI), followed by H_2_/PtO_2_ hydrogenation of intermediate iodides to convert them into hydrocarbons. Further analyses by tandem MS and GC/MS using supersonic molecular beam ionization revealed a series of hexamethylated derivatives **52**–**54.** At that, the methylation in peat membrane lipids was observed not only at position 5, but also at positions 6, 23, and 24 (Table 1) [89].

Adaptation of bacterial membrane lipids to temperature changes does not always lead to bipolar structures, while their adaptation to acidic environments, as a rule, requires bipolarity. The peculiarities of distribution of **43**–**54**, which are widely spread in natural sources, containing fresh waters, such as peats and surface soils, were used for reconstruction of paleotemperatures and of past soil pH through quantitative analysis of brGDGT fractions in 278 globally collected surface soil samples. Of nine brGDGTs found in soils, seven are even the most common and are additionally marked in Table 1 as **Ia**–**c, IIa**–**c**, and **IIIa**–**c [89]**.

Empirical relationships have also been established between indices of Methylation of Branched Tetraether (MBT and MBT’, calculated using data from only main 7 brGDGTs) as well as Cyclization of Branched Tetraether (CBT) indices and mean annual air temperatures (MAT) and soil pH. The relation between pH and CBT was found to be as follows: pH = 7.90–1.97 × CBT, whereas MAT correlated with both indices so that MAT = 0.81–5.67 × CBT + 31 × MBT’, where CBT = –Log (**Ib** + **IIb**)/(**Ia** + **IIa**) and MBT = (**Ia** + **Ib** + **Ic**)/(**Ia** + **Ib** + **Ic** + **IIa** + **IIb** + **IIc** + **IIIa** + **IIIb** + **IIIc**) [90].

brGDGTs are ubiquitous in sedimentary environments and well preserved in lacustrine sediments, which were used to analyze potential past continental temperatures. The advantage of brGDGTs as proxies in paleoclimatic studies, compared to archaeal *iso*-GDGTs, is that they can be used to analyze samples, collected not only from peat and soil but also from river and lake sediments. New distribution data obtained from a study of 91 samples of lacustrine surface sediments, collected from extensive tropical areas, including South America, East Africa, and Southeast Asia, showed a uniform response of lacustrine brGDGT compositions to mean annual air temperatures. By combining new data with those previously published, improved temperature calibrations were developed [91].

Recently, genes of homologs of archaeal tetraether synthase were identified in the genome of the bacterial strain *Candidatus Solibacter usitatus* Ellin6076 of an Acidobacterium. The diverse brGDGTs of this strain proved to contain C5-methylated and cyclic fragments. Culturing experiments demonstrated that the degree of C5-methylation in the bacterial strain is determined by temperature, while the degree of intrachain cyclization, leading to the formation of cyclopentane fragments in chains of bipolar lipids, depends on a number of factors, including temperature, pH, and oxygen availability [92].

**Scheme 1:**
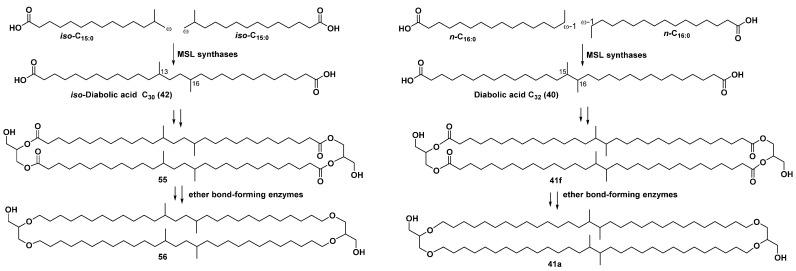
A simplified scheme of biosynthesis of brGDGTs in thermophilic bacteria, adopted from [93].

Biosynthesis of brGDGTs in bacterial producers was suggested to consist of the formation of dicarboxylic acid, such as **40**, **42** and related compounds, followed by esterification with glycerol and the formation of core bipolar lipids containing ester bonds and cyclopentane fragments with their subsequent transformation into ether-bonded membrane-spanning lipids (MSLs). Key enzymes responsible for the production of these lipids in some bacteria were recently identified. MSL synthases catalyze the dimerization of the FA building blocks through the formation of a C-C bond between either the ⍵-1 carbon atoms of *n*-C16:0 FA or between the ⍵ carbon atoms of *iso*-C15:0 FA, giving **40** or **42**, respectively. Actually, cultivation of the bacterium *Thermoanaerobacter ethanolicus* at 60 °C with ^13^C leucine in the medium led to the labeled *iso*-C15:0 FA, suggesting that *iso*-C15:0 FA is a probable biosynthetic precursor of **42**. Cultivation of the *T. maritima* at 80 °C led to **40** from *n*-palmitic acid (*n-*C16:0 FA) (Scheme 1). The search for genes encoding enzymes of this type was based on the suggestion that they are Fe-S cluster-associated SAM radical membrane proteins with a cysteine-rich motif (CxxxCxxC), earlier found in active sites of this type of enzymes, which are present in other MSL-producing archaea. Another type of enzyme, participating in the biosynthesis of bipolar MSLs, combines ether bond-forming proteins, whose genes were found in Thermotogae, various Acidobacteria, and Thermodesulfobacteria [93]. The biosynthetic transformations with participating such enzymes probably lead to the formation of the compounds **55** (and after it to **56)** from *iso*-diabolic acid (**42**) and compounds **41f** and **41a** from diabolic acid (**40**), respectively (Scheme 1).

In living bacterial cells, intact brGDGTs have polar groups attached to the terminal glycerol moieties of core lipids. Intact lipids of soil and peat have been studied much worse in comparison with their core lipids due to their lability under isolation conditions, since they are quickly destroyed after the lysis of bacterial cells, producing them. Intact lipids with hexose-glycuronic acid, phospho-hexose, and hexose-phosphoglycerol head groups were found and identified using HPLC/MS^2^ (HPLC/MS/MS) and HPLC/MS^3^ (MS/MS/MS) in samples collected from the Swedish peat bog Saxnäs Mosse. The fact that these brGDGTs were more abundant below the water table, supported the suggestion that they were produced mainly in oxygen-deprived parts of the peat [94].

In conclusion, it should be once again noted that bola-like membrane lipids of archaea and some bacteria show features of amazing structural similarities with each other. However, not only do terminal glycerol fragments in the corresponding metabolites from these Domains of microorganisms differ from each other stereochemically, but also their long hydrophobic chains linking these fragments to each other are biosynthesized in different ways: from isoprenoid precursors in archaea and from brFAs in some bacteria.

## 4. Sphingolipid-like Bolaamphiphiles from Marine Invertebrates

### 4.1. Sphingolipid-like Bolaamphiphiles from Sponges

Biochemical evolution of marine natural compounds led also to the emergence of other groups of bipolar bola-like natural products, found in some living systems, particularly in marine invertebrates. The corresponding sphingolipid-like compounds of marine invertebrates bear two distinctive polar termini, resembling «head» fragments of classical sphingolipids, and are linked to each other by a long hydrophobic chain. Moreover, they may contain an additional carbonyl group or another functional group in the chain. One of the polar fragments is usually glycosylated. It is unknown whether bioactive metabolites of this type are structural constituents of cellular membranes in their producers. Stereostructures and biological activities of these “bolaamphyphiles” are often unexpected, while their biological functions require further studies.

**Scheme 2:**
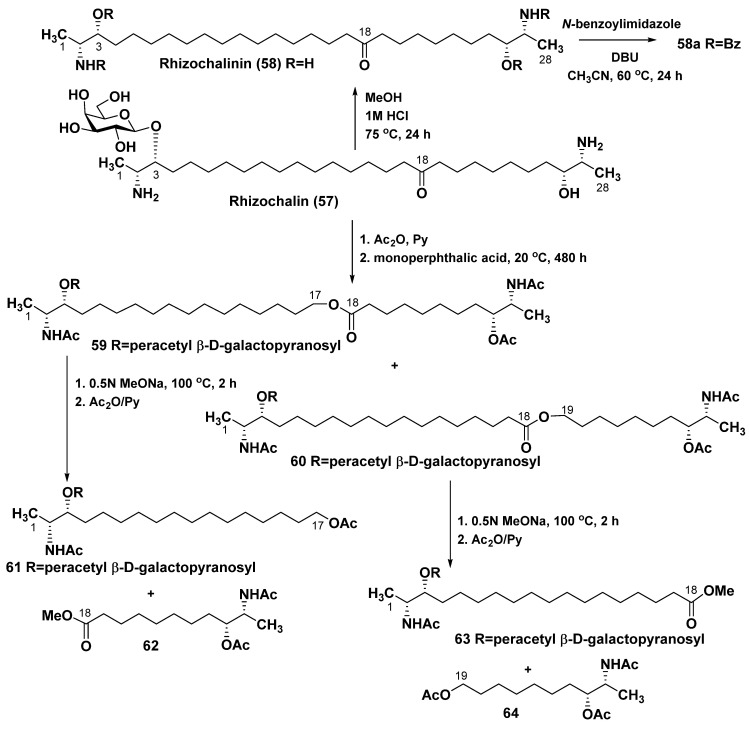
Determination of a keto group position in rhizochalin (**57**).

The first representative of this class of bipolar compounds was isolated by our group in 1989 from the sponge *Rhizochalina incrustata*, collected off the North coast line of Madagascar Island and named rhizochalin (**57**). (–)-Rhizochalin is a galactopyranoside, whose aglycon rhizochalinin (**58**) was obtained by methanolysis of **57** and later repeatedly studied in various biotests. In addition to difficulties of absolute configuration determination in its two termini, another problem concerned the establishing a position of a keto group in a long hydrocarbon chain of **57**. This problem was resolved using chemical transformations, NMR spectroscopy, and mass spectrometry. A mixture of **59** and **60**, having ester bonds on one or another side from a keto group, was obtained from rhizochalin peracetate by its oxidation with monoperphthalic acid (Baeyer–Villiger reaction) followed by acetylation. Alkaline hydrolysis of this mixture gave **61**–**64** as the products, whose MS and NMR spectra made it possible to determine the exact positions of substituents in rhizochalin [95] (Scheme 2).

The approach developed at that time was subsequently improved so that all procedures were carried out in one pot, while the replacement of monoperphthalic acid with hydrogen peroxide did not lead to contamination of the products, which allowed the use of small amounts of related natural compounds and ensured high yields of the target products [96]. This procedure was later applied to establish structures of other natural products belonging to the same structural group.

**Scheme 3:**
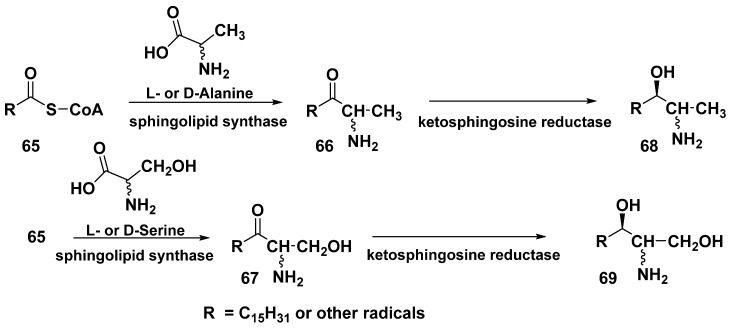
A hypothetic pathway of biosynthesis of terminal fragments in rhizochalin and related compounds.

Most likely, the terminal fragments in rhizoсhalin and its analogs are biosynthesized as a result of biochemical processes similar to those of sphingolipid biosynthesis, which consist in the action of sphingolipid synthase on acyl-CoAs (general formula **65**) at reaction the latter with serine or sometimes with alanine, which leads to the formation of intermediates **66** or **67**. The following conversions, catalyzed by ketosphingosine reductase, result in the biosynthesis of sphingolipids **68** and **69** (Scheme 3) [97]. One of the characteristic features of these conversions in some marine invertebrates is the participation of alanine instead of the more usual serine in this process, which leads to methyl-terminated sphingoids. The mechanism of coupling of the two sphingolipids, leading to the formation of rhizochalin and its analogs, as well as the corresponding enzymes, catalyzed this coupling, still remains unknown.

**Scheme 4:**
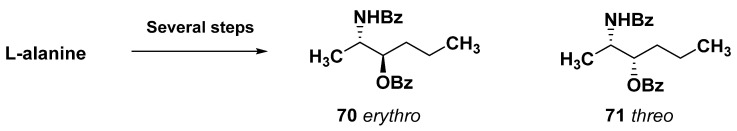
Synthesis of model compounds for determination of absolute configurations in rhizochalin.

Bipolar compounds of this type may be considered unusual pseudo-symmetric α,ω-bis-sphingoids with terminal groups remote from each other. Their absolute configurations were established by Molinski et al. [98]. To determine the absolute stereochemistry of **57** and **58**, the method of superposition in the CD spectra with exciton pairing (exciton-coupling CD (ECCD) superposition method) was developed. For this purpose, tetrabenzoyl derivative (**58a**) (Scheme 2) was obtained by the treatment of **58** with *N*-benzoylimidazole in the presence of 1,8-diazobicyclo[5.4.0]undecane (DBU). The obtained compound contains vicinal benzoate-benzamide pairs, which makes it possible to observe two Cotton effects in their CD spectra as a result of exciton interaction involving these groups. Stereoisomeric derivatives **70** and **71** were obtained by multistep synthesis from L-alanine and used as model compounds to analyze the CD spectrum of **58a** (Scheme 4).

The spectrum of **58a** was compared with the spectra of eight virtual hybrid molecules containing all the possible combinations of terminal groups with different stereochemistry. A good match was detected with the spectrum of one such molecule, namely a hybrid, having two *ent*-*threo* termini, that allowed for determining the 2*R*,3*R*,26*R*,27*R* absolute configurations in **58a** and, therefore, in rhizochalin (**57**). Surprisingly, these configurations were enantiomeric when compared with those of earlier known methyl-terminated sphingolipids. The 2*R*,3*R*,26*R*,27*R* stereochemistry was confirmed by COSY analysis and relatively large vicinal coupling constants (*J* = 5.5 Hz) of H-2/H-3 and H-26/H-27 in the ^1^H NMR spectrum [98].

Closely related bipolar metabolites were found in extracts of the sponges, collected off various geographical areas from the Cook Islands and Great Barrier Reef in the Pacific Ocean to the Seychelles and Tanzanian reefs in the Indian Ocean. In the corresponding papers, this type of natural product was sometimes called two-headed sphingolipids, although, strictly speaking, they do not belong to sphingolipids, but rather to sphingolipid-like metabolites. It is well known, that sphingolipids are not only important constituents of cellular membranes, but also biologically active substances, participating in the regulation of cell proliferation, differentiation, and apoptosis as extracellular and intracellular mediators. As was defined later, really bola-like metabolites of this group are able also to act as antagonists or agonists on some steps of sphingolipid biosynthesis.

Isorhizochalin (**72**) from the Indian Ocean sponge *Rhizochalina incrustata* was isolated as peracetate with a 2% yield from the fraction, in which the peracetate of **57** predominated [99]. Isorhizochalin was established to be a 2-epimer of **57** with absolute 2*S*,3*R*,26*R*,27*R* stereochemistry, supposing that its *α*-terminus is formed with the participation of D-alanine, while the ω-terminus of L-alanine. It was suggested that the rare D-alanine could be formed from L-isomer at the action of an epimerase from the sponge-producer or from a symbiont microorganism. Isorhizochalin exhibits cytotoxic effects against tumor HL-60 and THP-1 cells with IC_50_ of 2.9 and 2.2 µM [99].

**Figure 10:**
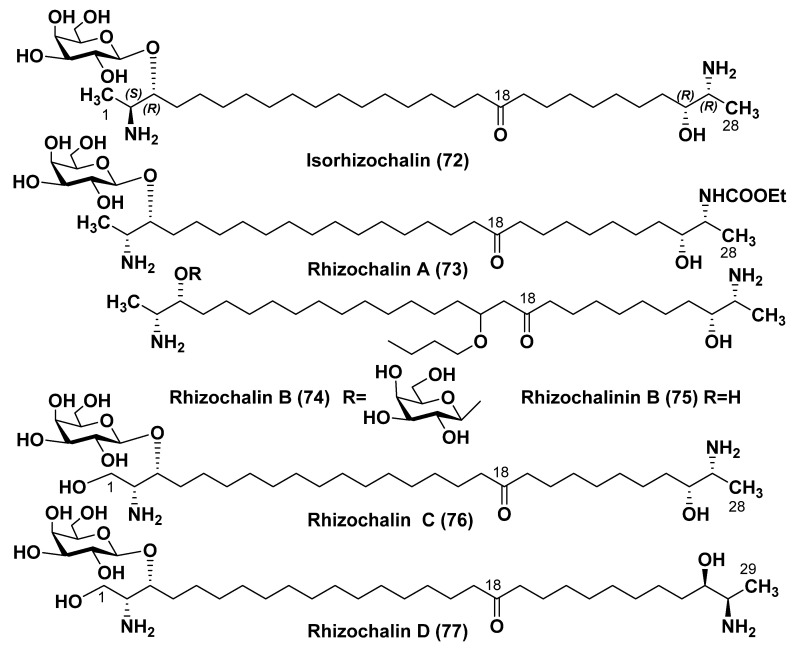
Sponge natural products **72**–**77**, related to rhizochalin.

Minor rhizochalin A (**73**) from the same sponge was also isolated as peracetate. Its structure was established by spectral methods and chemical transformations as 27-ethyl carbamate of **57** [100]. The related rhizochalin B (**74**) from a sponge *Oceanapia* sp. proved to be an artificial product, probably formed as a result of interaction with *n*-butanol, which was added to the sponge extract as a defoamer before concentration. This compound was accompanied by the corresponding aglycon—rhizochalinin B (**75**) [101]. Rhizochalin C (**76**) from the *R. incrustata* is related to rhizochalin in its *threo*-configurations at both termini, but one of them was formed with the participation of serine instead of alanine [96]. Rhizochalin D (**77**), isolated together with **57**, proved to be its homolog containing a chain of 29 carbon atoms, in contrast to all other compounds of this series, containing one less carbon atom [96] (Figure 10).

Rhizochalin (**57**) and related compounds inhibit the growth of *Staphylococcus aureus* and demonstrate cytotoxicity against various tumor cells via the induction of P53-dependent apoptosis with activation of caspases, even in castration-resistant prostate cancer cells. This metabolite reduces the expression of the alternative splicing variant of the androgen receptor AR-V7 in these cells, thereby increasing their sensitivity to antitumor drugs. Rhizochalinin (**58**) is the most active in a series of the studied bipolar compounds, showing the apoptosis of HT-29 cancer cells is realized through AMP-activated protein kinase [102,103,104]. This compound is also a potential cancer preventive agent because it inhibits the EGF-induced malignant transformation and colony formations in JB6 P^+^ Cl 41 cells [105]. In addition, rhizochalinin acts on spermatozoa of the sea urchin *Strongylocentrotus intermedius*, inducing their apoptosis. It is possible that the Ca^2+^, Mg^2+^-dependent sea urchin DNAase participates in this process because no apoptosis was detected after the pretreatment of spermatozoa with a specific inhibitor of this enzyme (Zn^2+^ ions) [106].

**Figure 11:**
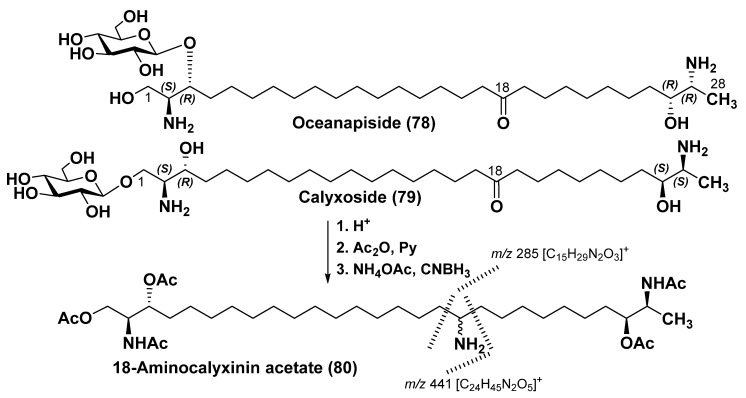
Structures of oceanapiside (**78**) and calyxoside (**79**).

One more bolaamphyphilic natural product of the same series, oceanapiside (**78**), was isolated from the sponge *Oceanаpia phillipensis*, collected from the Australian Port Phillip Bay. It contains a β-D-glucopyranose residue instead of galactopyranose, which is more common in metabolites belonging to this structural group. The position of the keto group in its long hydrophobic chain was also determined by a procedure based on Baeyer–Villiger oxidation [96,107,108]. The complete structure of **78** (Figure 11) was established by 2D NMR spectroscopy, degradation methods, and tandem mass spectrometry. The absolute stereochemistry was established as 2*S*,3*R*,26*R*,27*R* from the CD spectra of the perbenzoyl derivative of **78** using the above-mentioned ECCD superposition method. Compound **78** contains the *erythro*- and *threo*-configurations at positions 2,3 and 26,27, showing that enantiodivergent biosynthesis of this unusual metabolite takes place with the formal participation of L-serine and a rare D-alanine [109]. Oceanapiside exhibits an inhibitory effect against the pathogenic fluconazole-resistant microscopic fungus *Candida glabrata* with IC_50_ of 10 µg/mL, affecting the morphology of fungal cells and interfering with the biosynthesis of sphingoids in this pathogen. It increases the proportion of phytosphinganine bases as a result of the inhibition of their transformation into phytoceramides in the fungus. Thus, the mechanism of inhibitory effect of **78** against *C. glabrata* is quite different in comparison with that of the previously known antifungal antibiotics amphotericin B and miconazole [110].

A sponge *Calyx* sp., collected off the Palau reefs in the Pacific Ocean, contains the bola-like glycoside calyxoside (**79**). It was isolated by Zhou et al. as a result of the search for new marine natural agents that damage DNA. The position of the carbonyl group in **79** was determined by obtaining its aglycon followed by acetylation and replacing this group with an amino group by hydroamination at treatment with lithium cyanoborohydride and ammonium acetate to obtain 18-aminocalyxinin peracetate (**80**). Subsequent MS analysis gave the corresponding diagnostic fragmentation, as shown in Figure 11. The absolute configuration of calyxoside was determined as 2*S*,3*R*,26*S*,27*S* using NMR and CD spectroscopy of calyxoside aglycon (calyxinin) tetrabenzoate [111].

**Scheme 5:**
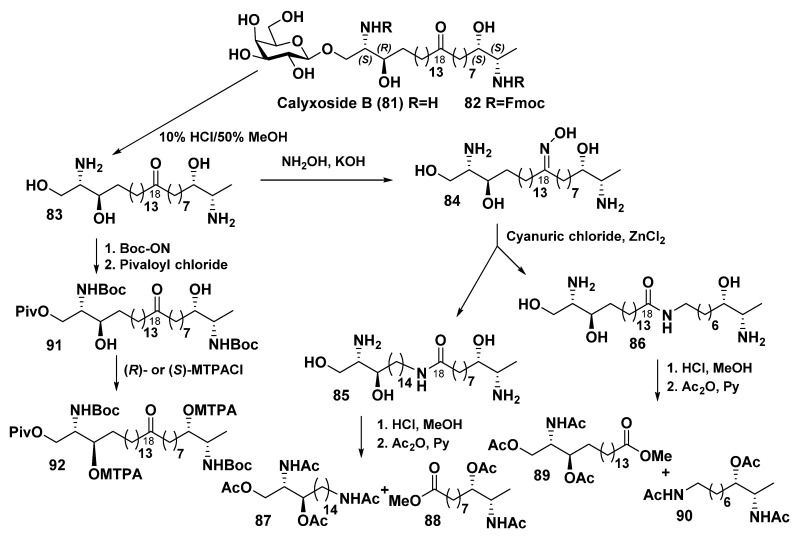
Chemical transformations of calyxoside B (**81**) [112].

The related calyxoside B (**81**) was isolated from a deep-sea sponge *Cladocroce* sp., collected by dredging from the depth of 245 m at the Gonsone seamount during a marine expedition onboard the R/V “Nagasaki-Maru” (Scheme 5). It was shown to be a β-D-galactopyranosyl analog of calyxoside. Its structure elucidation was difficult because, on the first step of the study, an inseparable mixture of **79** and **81** was isolated. Only after obtaining fluoromethyloxycarbonyl (Fmoc) derivative, the corresponding individual compound was purified by recycled HPLC with 20 cycles. The Fmoc derivative **82** was converted into calyxoside B itself (**81**) by removal of the protective group. Aglycon of calyxoside B (**83**) was isolated after acid hydrolysis and converted to oxime **84**. The subsequent Beckmann rearrangement gave two isomeric amides (**85** and **86**), subjected to acid hydrolysis and acetylation to afford products **87**–**90**. Their LC/MS examination made possible the determination of a keto group position within a long hydrocarbon chain of parent bola-like compound **81**. It turned out to be the same as in other bipolar metabolites of this structural series.

**Scheme 6:**
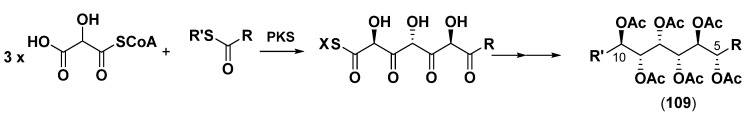
Hypothetical biosynthetic pathway to hexa-*O*-acetylated fragment (**109**) in sagittamides (adopted from [124]).

The absolute configurations of the asymmetric centers in the two terminal fragments of calyxoside B were determined by chemical conversions of the aglycon **83** (Scheme 6). At that, each of the amino groups in **83** was protected by *t*-butoxycarbonylation with Boc-ON, while the primary hydroxyls were converted into *O*-pivaloyl groups (*O*-Piv) to afford the derivative **91**, containing a free secondary hydroxy group, that allowed for obtaining Mosher’s derivatives **92** and determining of the absolute configurations as 2*S*,3*R*,26*S*,27*S* by NMR (Scheme 5) [112].

**Figure 12:**
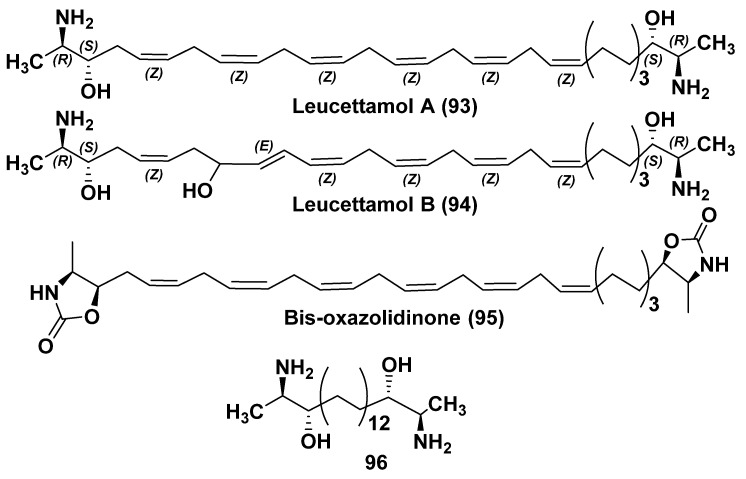
Structures of leucettamols A (**93**) and B (**94**) and some derivatives of **93**.

As a result of the screening for antimicrobial metabolites, which inhibit the pathogenic bacterium *Bacillus subtilis*, Kong and Faulkner found and isolated unusual bipolar long-chain polyunsaturated diaminodiols leucettamols A (**93**) and B (**94**) from the calcareous sponge *Leucetta microraphis*, collected off the Palau Islands [113]. These compounds also belong to the bola-like metabolites of sponges. The authors initially reported that **93** is a racemate, but it was not confirmed by subsequent examination. The relative configurations in terminal 2-amino-3-hydroxy structural fragments were established by NMR spectroscopy using the nuclear Overhauser effects in bis-oxazolidinone (**95**), obtained by the reaction of leucettamol A with 1,1′-diimidazole (Figure 12). The absolute configurations of leucettamol A (**93**) were determined by its conversion to the *N,N’,O,O’*-tetrabenzoyl derivative (followed by an examination of the ECCD spectrum. This CD approach unambiguously established that **93** is a chiral natural product having optically active pseudo-*C*_2_ symmetry. The stereostructure of each terminus proved to be *erythro* with 2*R*,3*S*,28*S*,29*R* absolute configurations. The application of ECCD to establish absolute configurations of all stereogenic centers provided greater sensitivity compared to ^1^H NMR *J*-based methods [114].

Being the first natural inhibitor of Ubc13-Uev1A interaction, leucettamol A (**93**) inhibits the formation of ubiquitin conjugating complex of Ubc13 and Uev1A tumor promoter with IC_50_ value of 50 μg/mL [115]. This compound was also shown to be a leukotriene B4 receptor antagonist. Inhibitors of this receptor are promising for the creation of anticancer and antirheumatoid drugs. Leucettamols and their semisynthetic derivatives, such as perhydro derivative (**96**), were shown to be TRP ion channel modulators. TRP channels were indicated in the plasmatic membranes of animals and in other eukaryotes and serve as biosensors that regulate membrane permeability to calcium and magnesium ions in response to such stimuli as pain, fever, and touch [116]. Disturbances in the functioning of such channels are the cause of a number of diseases. At that, the activity of **96** towards the channels was about 10 times higher compared to that of leucettamol A itself. Substances of this series act as non-electrophilic activators of TRPA1 channels, inhibiting the activity of TRPM8 channels [117].

### 4.2. Bipolar Alkaloidolipids from Sponges

**Figure 13:**
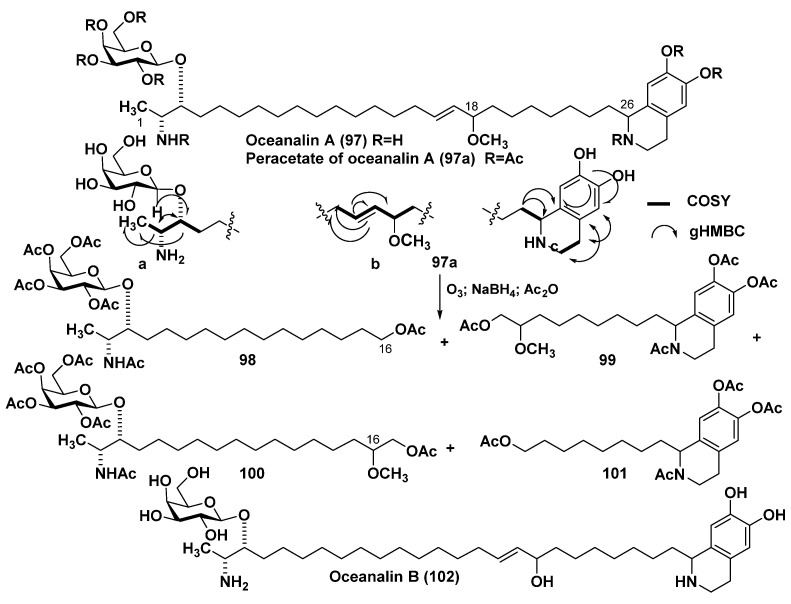
Oceanalins A (**97**) and B (**102**) and some derivatives of oceanalin A (**98–101**).

Among the bipolar metabolites of sponges, there is a separate group, consisting of unprecedented alkaloidolipids called oceanalins. The first of them, oceanalin A (**97**), was isolated from ethanolic extract of a sponge *Oceanapia* sp. collected near the Scott Reef off the northwest coast of Australia and proved to be α,ω-bifunctional hybrid, in which a sphingolipid-like moiety and a dihydroxytetrahydroisoquinoline fragment are connected to each other by a long hydrophobic chain. NMR spectra revealed the subfragments **a**, **b,** and **c**, while ozonolysis of peracetate **97a** after reductive treatment gave compounds **98** and **99**, accompanied by **100** and **101**, which were formed from an impurity of the isomeric product obtained as a result of allyl rearrangement of oceanalin A. The hydrolysis of **97** with 6 N HCl gave D-galactose. The 2*R*,3*R* configurations of the corresponding stereogenic centers were established by interpretation of the CD spectra of the benzoyl derivative and turned out to be the same as in rhizochalin (Figure 13). Spontaneous isomerization at C-26 took place in the isoquinoline fragment of **97**, and, therefore, this compound and its derivatives were shown to be the mixtures (1:1) of two C-26 epimers. Oceanalin A exhibits an inhibitory effect against the flucanazole-resistant fungus *Candida glabrata* with MIC of 30 µg/mL as a result of blocking the biosynthesis of phytosphingolipids in this pathogen [118].

Oceanalin B (**102**) from the same sponge is the second representative of this unusual group of hybrid bolaamphiphilic natural products isolated from *Oceanapia* sp. (Figure 13). It was established to be a hydroxy derivative of **97** and probably this more labile compound is really a genuine natural product, while **97** is an artificial product, which is formed from **102** during its isolation using methanol. Oceanalin B turned out to be more active (MIC 25 µg/mL) than **97** against the drug-resistant fungus *Candida glabrata* [119].

### 4.3. Sagittamides

**Figure 14:**
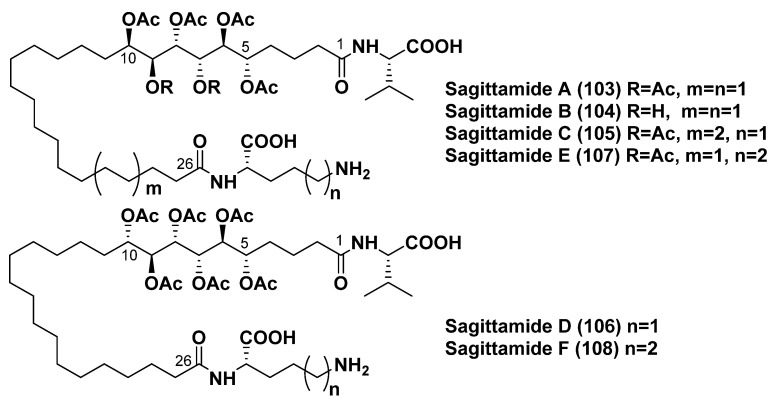
Sagittamides A–F (**103**–**108**).

Among marine invertebrates, biological sources of two-headed metabolites are not limited to only sponges. Another taxonomic group, in which such substances were found, is represented by ascidians (phylum Chordata, class Ascidiacea). Sagittamides, belonging to a unique group of this type of compounds with atypically modified hydrophobic chains and polar termini containing amino acid residues, were discovered by Lievens and Molinski, who isolated them from an unidentified didemnid ascidian collected off the Pohnpei Island (Micronesia). The first two members of this series, sagittamides A (**103**) and B (**104**), contain in their structures unbranched C_26_ dicarboxylic acid linked by amide bonds to the terminal amino acid residues of L-valine and L-ornithine (Figure 14) [120]. A surprising structural peculiarity of these acids consists of the presence of unprecedented internal *O*-hexaacetyl-1,2,3,4,5,6-hexaol fragments in their long chains, which made elucidation the structures and, in particular, determination of the stereochemistry of **103** and **104** quite difficult [119]. For the first attempt using an approach integrating three modes of stereochemical analysis (*J*-based analysis, ^13^C NMR database, and exciton coupling CD), the absolute configuration of the *O*-hexaacetyl hexahydroxy-α,ω-dicarboxylic long-chain motif in **103** and **104** was determined to have *anti-anti-anti-syn-anti* configuration [121]. However, some later, Seike et al. made an independent assignment of *anti-anti-syn-anti-syn* configuration for the C5–C10 segment in **103** based on a comparison of obtained NMR data of two synthesized diastereomeric compounds with those of the natural product [122]. Additional analysis unambiguously confirmed the latter configuration using carefully remeasured ^2^*J*_CH_ and ^3^*J*_CH_ NMR constants, NOEs, and residual dipolar coupling enhanced NMR [123].

Minor sagittamides C–F (**105**–**108**) were isolated from the same ascidian and studied by NMR spectroscopy and comparison of the obtained data with those of **103** as well as by degradation to amino acids. Sagittamide C (**105**) contains two additional peaks of CH_2_ groups in the ^13^C NMR spectrum compared to the spectrum of **103** and has a longer C_28_ α,ω-dicarboxylated chain with the same configuration in the C-5–C-10 fragment. Sagittamides D (**106**) and F (**108**) were established to possess *anti-anti-syn-anti-anti* configuration in the inner hexaacetoxy fragment with an inversion of C-10 stereochemistry in comparison with **103**, while E (**107**) and F (**108**) differed from **103** in the terminal amino acid (L-lysine instead of L-ornithine) (Figure 14) [124].

Biosynthesis of the unusual hexa-*O*-acetyl segment (**109**) in sagittamides was not studied, but it was suggested that it consists of either post-translational oxidative modification of the long chain of the parent dicarboxylic acid or in the biosynthesis of the C-5–C-10 fragment by polyketide synthase (PKS) with the participation of three hydroxymalonyl CoA units as substrates (Scheme 6) [124].

The significant difficulties, associated with the structure elucidation of such complex natural products stimulate the development of special approaches, including the application of organic partial or total syntheses. One more model compound was recently synthesized that should help establish the absolute stereochemistry of more complex structures of so far undiscovered sagittamides [125].

It is noteworthy that many bola-like secondary metabolites of marine invertebrates are characterized by a number of the same structural features as similar metabolites of bacterial origin, for example, by bipolarity and predominately C_26_-C_28_ lengths of main hydrophobic chains. At the present time, nothing is known about the possible participation of symbiotic microorganisms in their formation in ascidians and sponges.

## 5. Other Bola-like Metabolites from Sponges

Some other groups of bipolar marine metabolites are also known, for example, the bioactive steroid dimers from sponges such as fibrosterol sulfates [126], manadosterols [127], and others, formed by coupling of two polysulfated steroids via side chains. However, the resulting chains in these compounds are not long and flexible enough to classify these metabolites as bola-like metabolites.

On the contrary, a group of α,ω-bifunctionalized derivatives of long-chain fatty acids, which are amidated by residues of tyramine or dopamine at the α-terminus and glycosylated by glucuronic acid at the ω-terminus, might well be classified as bola-like natural products. These compounds were simultaneously discovered in 2016 from the sponges inhabiting the Northern waters of the World Ocean by two groups of scientists, an Icelandic group from the University of Iceland, Reykjavik, and our group from the Pacific Institute of Bioorganic Chemistry, Vladivostok, Russia.

**Figure 15:**
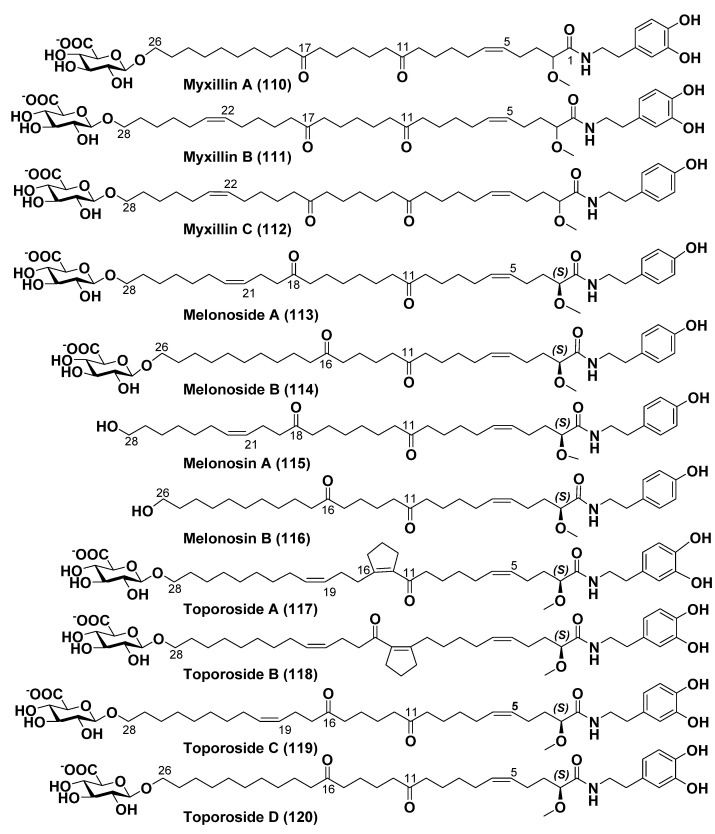
Structures of α,ω-bifunctionalized bola-like metabolites **110**–**120** from some sponges.

Icelandic scientists have found and isolated this novel group of bipolar lipids, named myxillins A–C (**110**–**112**), from the sponge *Myxilla incrustans* collected in the area of a submarine hydrothermal vent Strytan, North of Iceland (Figure 15). Their structures were determined by HRMS, 1D, and 2D NMR spectroscopy. However, configurations of an asymmetric center at C-2 in these bipolar lipids were not established. Myxillins A (**110**) and C (**112**) showed immunomodulatory action in an in vitro dendritic cell model. At that, **110** at the concentration of 10 μg/mL lowered levels of IL-12p40, suggesting a potential anti-inflammatory effect, while **112** at the same dose lowered levels of cytokine IL-10, suggesting a potential pro-inflammatory action [128].

Russian scientists isolated a related ω-glycosylated fatty acid amide, named melonoside A (**113**), from the deep-water marine sponge *Melonanchora kobjakovae,* collected in the Sea of Okhotsk near Urup Island (Figure 15). Their collaborators from the University Medical Center Hamburg-Eppendorf, Germany, showed that **113** at the dose of 10 μM induces autophagy of human cisplatin-resistant germinal tumor cells NCCIT-R. The structure of **113**, including the exact location of keto groups and double bonds, was unambiguously determined using not only MS and 2D NMR experiments but also chemical conversions, including ozonolysis, hydroamination, acid hydrolysis, followed by analysis of the obtained products by HRESIMS. The absolute configuration of the C-2 stereogenic center was established by comparison of CD spectra of the aglycon of melonoside A (so-called melonosin A (**115**)) and a series of model compounds [129].

Sometime later, the related melonoside B (**114**) along with melonosins A (**115**) and B (**116**) were isolated from another collection of the sponge* M. kobjakovae *(Figure 15). Melonosins showed the inhibition of AP-1- and NF-kB-dependent transcriptional activities in JB6 Cl41 cells at noncytotoxic concentrations, as it is characteristic of potential cancer preventive agents [130].

Structures, including absolute configurations, of toporosides A–D (**117**–**120**) (Figure 15) from the collected in the Northwestern Pacific sponge *Stelodoryx toporoki*, were determined by 1D and 2D NMR, HRESIMS, chemical transformations, and comparison with their ECD spectra with those of synthetic model compounds. Toporosides A, C, and D at the concentration of 2.5 µM showed protective action against TNF-α-induced damage of H9c2 cardiomyocytes. The interesting structure peculiarity of **117** and **118** consists of the presence of five-membered rings in their chains, probably formed as a result of intamolecular aldol condensation in **119** [131].

Thus, marine invertebrates, particularly sponges, demonstrate an impressive structural variety of bola-like metabolites, but the majority have the same length of chains connecting their polar “heads”. These natural products differ from each other in polar fragments and inclusion within their long chains of different groups, frequently ketones, double bonds, and even polyhydroxylated fragments. Many questions, concerning their biogenesis and biological functions, still remain unanswered.

Some of the bolaamphipilic natural products, reviewed by us, are prospects for medical application (Table 2), although the majority of bioactive bolaamphiphilic compounds are rather synthetic than natural compounds [1,132,133,134].

Recently, artificial gel-forming bolalipids were proposed as new formulations in atimicrobial and antifungal therapy [135].

Prospects for further study of bola-like natural biomolecules include the search for new compounds of this class, studies on structural features and bioactivities, an expansion of their practical application in experimental medicine and biotechnology, and, finally, the use and detailed study of their still insufficiently studied physicochemical properties. However, not all these areas fit into the framework of the concept of this review article, which is primarily devoted to the structural diversity and biological activity of different representatives of this unique class of biomolecules.

## 6. Conclusions

A great structure diversity of natural bola-like metabolites provides the clue to understanding the principles of adaptation of their producers to harsh environmental conditions. However, some structural features of these substances, particularly, stereochemistry, continue to remain poorly studied. Main directions of modification of core structures in bola-like metabolites are realized by a great variety of bioconversions such as alkylation of terminal glycerol moieties, inclusion of cyclopentane and cyclohexane fragments, as well as additional introduction of methyl, carbonyl, and hydroxy groups into their long chains. Polar termini of many natural bolaamphiphiles are probably constructed in the same manner as in convenient lipids with the participation of glycerol or other lipids, for example, sphingoids in bola-like metabolites of some marine invertebrates. The comparative structural analysis of bola-like natural products, discussed in this review, confirms the known thesis that nature does not forget its successful findings (such as the bipolarity of some metabolites and the presence of long and flexible hydrocarbon chains, connecting polar termini to each other, in them), but repeats them over and over again at various taxonomic levels. The obtaining of individual natural bolaamphiphiles and their stereochemical analysis represent challenges for structure studies on such natural products. For instance, this was shown by the example of the seemingly well-studied crenarchaeols, the structures of which were revised after applying rather complex syntheses to this type of substance.

## Figures and Tables

**Table 1:** Some brGDGTs from peat and soil.

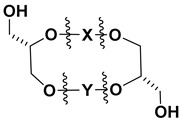		
**Number**	**Side Chains (X, Y)**	**Ref** **s** **.**
**43 (Ia)**	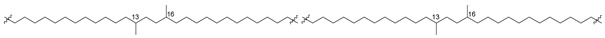	[87,88]
**44 (Ib)**	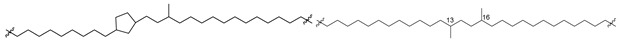	[87,88]
**45 (Ic)**	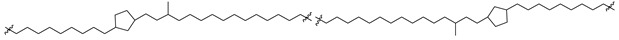	[87,88]
**46 (IIa)**	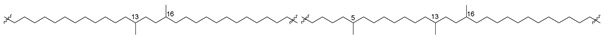	[87,88]
**47 (IIb)**	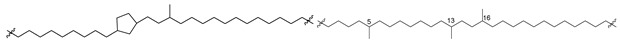	[87,88]
**48 (IIc)**	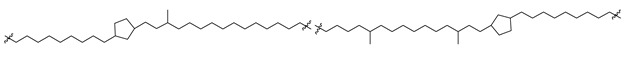	[87,88]
**49 (IIIa)**	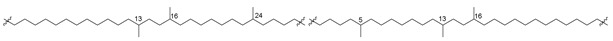	[87,88]
**50 (IIIb)**	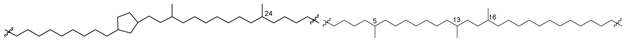	[87,88]
**51 (Шс)**	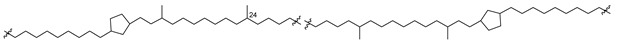	[87,88]
**52**	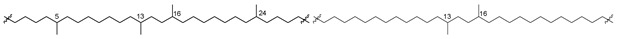	[89]
**53**	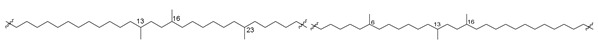	[89]
**54**	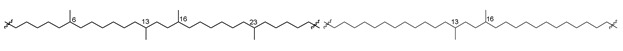	[89]

**Table 2:** Bioactivity of some bola-like natural compounds.

Bioactivity	Compound	Refs.
Anticancer	rhizochalin (**57**)	[102]
	rhizochalinin (**58**)	[104]
	isorhizochalin (**72**)	[99]
	melonoside A (**113**)	[129]
Antifungal	oceanapiside (**78**)	[108]
	oceanalin A (**97**)	[118]
	oceanalin B (**102**)	[119]
Antimicrobial	leucettamol A (**93**)	[113]
	leucettamol B (**94**)	
Cancer preventive	melonosin A (**115**)	[130]
	rhizochalin (**57**)	[105]
Cardioprotective	toporoside A (**117**)	[131]
	toporoside C (**119**)	
	toporoside D (**120**)	
Drug delivery system	caldarchaeol (**3**)	[73,74,75,76,77,78]
	GDGTs	
	crenarchaeol (**14**)	
Inhibitor of Ubc13-Uev1A interaction	leucettamol A (**93**)	[115]
Immunomodulatory	myxillin A (**110**)	[128]
	myxillin C (**112**)	
Modulators of TRPA1 and TRPM8 channels	leucettamol A (**93**)	[117]
	leucettamol B (**94**)	

## Data Availability

All relevant data are included in the manuscript.

## References

[1] Fariya M., Jain A., Dhawan V., Shah S., Nagarsenker M.S. (2014). Bolaamphiphiles: A pharmaceutical review. Adv. Pharm. Bull..

[2] Woese C.R., Fox G.E. (1977). Phylogenetic structure of the prokaryotic domain: The primary kingdoms. Proc. Natl. Acad. Sci. USA.

[3] Woese C.R., Kandler O., Wheelis M.L. (1990). Towards a natural system of organisms: Proposal for the domains Archaea, Bacteria, and Eucarya. Proc. Natl. Acad. Sci. USA.

[4] Wang M., Yafremava L.S., Caetano-Anolles D., Mittenthal J.E., Caetano-Anolles G. (2007). Reductive evolution of architectural repertoires in proteomes and the birth of the tripartite world. Genome Res..

[5] Kim J.-G., Jung M.-Y., Park S.-J., Rijpstra W.I.C., Sinninghe Damsté J.S., Madsen E.L., Min D., Kim J.-S., Kim G.-J., Rhee S.-K. (2012). Cultivation of a highly enriched ammonia-oxidizing archaeon of thaumarchaeotal group I.1b from an agricultural soil. Environ. Microbiol..

[6] Xia X., Guo W., Liu H. (2017). Basin scale variation on the composition and diversity of Archaea in the Pacific Ocean. Front. Microbiol..

[7] Santoro A.E., Richter R.A., Dupont C.L. (2019). Planktonic marine archaea. Ann. Revs. Mar. Sci..

[8] Lincoln S.A., Wai B., Eppley J.M., Church M.J., Summons R.E., DeLong E.F. (2014). Planktonic Euryarchaeota are a significant source of archaeal tetraether lipids in the ocean. Proc. Natl. Acad. Sci. USA.

[9] Karner M.B., DeLong E.F., Karl D.M. (2001). Archaeal dominance in the mesopelagic zone of the Pacific Ocean. Nature.

[10] Lipp J.S., Morono Y., Inagaki F., Hinrichs K.-U. (2008). Significant contribution of Archaea to extant biomass in marine subsurface sediments. Nature.

[11] Moissl-Eichinger C., Pausan M., Taffner J., Berg G., Bang C., Schmitz R.A. (2018). Archaea are interactive components of complex microbiomes. Trends Microbiol..

[12] Volmer J.G., McRae H., Morrison M. (2023). The evolving role of methanogenic archaea in mammalian microbiomes. Front. Microbiol..

[13] Wrede C., Dreier A., Kokoschka S., Hoppert M. (2012). Archaea in symbioses. Archaea.

[14] Moissl-Eichinger C., Huber H. (2011). Archaeal symbionts and parasites. Curr. Opin. Microbiol..

[15] Koga Y. (2014). From promiscuity to the lipid divide: On the evolution of distinct membranes in Archaea and Bacteria. J. Mol. Evol..

[16] Kruger M., Meyerdierks A., Glockner F.O., Amann R., Widdel F., Kube M., Reinhardt R., Kahnt J., Böcher R., Thauer R.K. (2003). A conspicuous nickel protein in microbial mats that oxidize methane anaerobically. Nature.

[17] Takano Y., Kaneko M., Kahnt J., Imachi H., Shima S., Ohkouchi N. (2013). Detection of coenzyme F430 in deep sea sediments: A key molecule for biological methanogenesis. Org. Geochem..

[18] Kates M., Yengoyan L.S., Sastry P.S. (1965). A diether analog of phosphatidyl glycerophosphate in *Halobacterium cutirubrum*. Biochim. Biophys. Acta.

[19] Langworthy T.A. (1977). Long-chain diglycerol tetraethers from *Thermoplasma acidophilum*. Biochim. Biophys. Acta.

[20] Gräther O., Arigoni D. (1995). Detection of regioisomeric macrocyclic tetraethers in the lipids of Methanobacterium thermoautotrophicum and other archaeal organisms. J. Chem. Soc. Chem. Comm..

[21] DeRosa M., Gambacorta A., Nicolaus B., Sodano S., Bu’Lock J.D. (1980). Structural regularities in tetraether lipids of Caldariella and their biosynthetic and phyletic implications. Phytochem.

[22] DeRosa M., Gambacorta A., Gliozzi A. (1986). Structure, biosynthesis, and physicochemical properties of archaebacterial lipids. Microbiol. Rev..

[23] Hanford M.J., Peeples T.L. (2002). Archaeal tetraether lipids: Unique structures and applications. Appl. Biochem. Biotechnol..

[24] Chong P.L.-G. (2010). Archaebacterial bipolar tetraether lipids: Physico-chemical and membrane properties. Chem. Phys. Lipids.

[25] Pearson A. (2019). Resolving a piece of the archaeal lipid puzzle. Proc. Natl. Acad. Sci. USA.

[26] Řezanka T., Kyselová L., Murphy D.J. (2023). Archaeal lipids. Prog. Lipid Res..

[27] Zeng Z., Chen H., Yang H., Chen Y., Yang W., Xi F., Pei X., Welander P.V. (2022). Identification of a protein responsible for the synthesis of archaeal membrane-spanning GDGT lipids. Nat. Commun..

[28] Lloyd C.T., Iwig D.F., Wang B., Cossu M., Metcalf W.W., Boal A.K., Booker S.J. (2022). Discovery, structure and mechanism of a tetraether lipid synthase. Nature.

[29] Eguchi T., Ibaragi K., Kakinuma K. (1998). Total synthesis of archaeal 72-membered macrocyclic tetraether lipids. J. Org. Chem..

[30] Andringa R.L.H., de Kok N.A.W., Driessen A.J.M., Minnaard A.J. (2021). A unified approach for the total synthesis of *cyclo*-archaeol, *iso*-caldarchaeol, caldarchaeol, and mycoketide. Angew. Chem..

[31] Sita L.R. (1993). Convenient highly stereoselective syntheses of (3R, 7R, 11R)-and (3S, 7R, 11R)-3, 7, 11, 15-tetramethylhexadecanoic acid (phytanic acid) and the corresponding 3, 7, 11, 15-tetramethylhexadecan-1-ols. J. Org. Chem..

[32] Wang A., Wüstenberg B., Pfaltz A. (2008). Enantio- and diastereoselective hydrogenation of farnesol and O-protected derivatives: Stereocontrol by changing the C=C bond configuration. Angew. Chem..

[33] Sinninghe Damsté J.S., Schouten S., Hopmans E.C., van Duin A.C.T., Geenevasen J.A.J. (2002). Crenarchaeol: The characteristic core glycerol dibiphytanyl glycerol tetraether membrane lipid of cosmopolitan pelagic crenarchaeota. J. Lipid Res..

[34] Takano Y., Chikaraishi Y., Ogawa N.O., Nomaki H., Morono Y., Inagaki F., Kitazato H., Hinrichs K.-U., Ohkouchi N. (2010). Sedimentary membrane lipids recycled by deep-sea benthic archaea. Nat. Geosci..

[35] Knappy C.S., Chong J.P., Keely B.J. (2009). Rapid discrimination of archaeal tetraether lipid cores by liquid chromatography-tandem mass spectrometry. J. Am. Soc. Mass Spectrom..

[36] Knappy C.S., Barillà D., De Blaquiere J.P.A., Morgan H.W., Nunn C.E.M., Suleman M., Tan C.H.W., Keely B.J. (2012). Structural complexity in isoprenoid glycerol dialkyl glycerol tetraether lipid cores of *Sulfolobus* and other archaea revealed by liquid chromatography-tandem mass spectrometry. Chem. Phys. Lipids.

[37] Blѐriot Y., Untersteller E., Fritz B., Sinaÿ P. (2002). Total synthesis of calditol: Structural clarification of this typical component of Archaea order Sulfolobales. Chem. A Eur. J..

[38] Siliakus M.F., van der Oost J., Kengen S.W.M. (2017). Adaptations of archaeal and bacterial membranes to variations in temperature, pH and pressure. Extremophiles.

[39] Zeng Z., Liu X.-L., Wei J.H., Summons R.E., Welander P.V. (2018). Calditol-linked membrane lipids are required for acid tolerance in *Sulfolobus acidocaldarius*. Proc. Natl. Acad. Sci. USA.

[40] Zeng Z., Liu X.-L., Farley K.R., Wei J.H., Metcalf W.W., Summons R.E., Welander P.V. (2019). GDGT cyclization proteins identify the dominant archaeal sources of tetraether lipids in the ocean. Proc. Natl. Acad. Sci. USA.

[41] Holzheimer M., Sinninghe Damsté J.S., Schouten S., Havenith R.W.A., Cunha A.V., Minnaard A.J. (2021). Total synthesis of the alleged structure of crenarchaeol enables structure revision. Angew. Chem..

[42] Sinninghe Damsté J.S., Rijpstra W.I.C., Hopmans E.C., den Uijl M.J., Weijers J.W.H., Schouten S. (2018). The enigmatic structure of the crenarchaeol isomer. Org. Geochem..

[43] Summons R.E., Welander P.V., Gold D.A. (2022). Lipid biomarkers: Molecular tools for illuminating the history of microbial life. Nat. Rev. Microbiol..

[44] Schouten S., Hopmans E.S., Sinninghe Damsté J.S. (2013). The organic geochemistry of glycerol dialkyl glycerol tetraether lipids: A review. Org. Geochem..

[45] Schouten S., Hopmans E.C., Schefuß E., Sinninghe Damsté J.S. (2002). Distributional variations in marine crenarchaeotal membrane lipids: A new tool for reconstructing ancient sea water temperatures?. Earth Planet. Sci. Lett..

[46] Kim J.-H., van der Meer J., Schouten S., Helmke P., Willmott V., Sangiorgi F., Koc N., Hopmans E.C., Sinninghe Damsté J.S. (2010). New indices and calibrations derived from the distribution of crenarchaeal isoprenoid tetraether lipids: Implications for past sea surface temperature reconstructions. Geochim. Cosmochim. Acta.

[47] Ho S.L., Mollenhauer G., Fietz S., Martínez-Garcia A., Lamy F., Rueda G., Schipper K., Meheust M., Rosell-Mele A., Stein R. (2014). Appraisal of TEX86 and TEX86L thermometries in subpolar and polar regions. Geochim. Cosmochim. Acta.

[48] Fietz S., Ho S.L., Huguet C. (2020). Archaeal membrane lipid-based paleothermometry for applications in polar oceans. Oceanography.

[49] Morii H., Eguchi T., Nishihara M., Kakinuma K., König H., Koga Y. (1998). A novel ether core lipid with H-shaped C_80_-isoprenoid hydrocarbon chain from the hyperthermophilic methanogen *Methanothermus fervidus*. Biochem. Biophys. Acta.

[50] Schouten S., Baas M., Hopmans E.C., Reysenbach A.-L., Sinninghe Damsté J.S. (2008). Tetraether membrane lipids of *Candidatus* “*Aciduliprofundum boonei*”, a cultivated obligate thermoacidophilic euryarchaeote from deep-sea hydrothermal vents. Extremophiles.

[51] Zhu C., Meador T.B., Dummann W., Hinrichs K.-U. (2014). Identification of unusual butanetriol dialkyl glycerol tetraether and pentanetriol dialkyl glycerol tetraether lipids in marine sediments. Rapid Commun. Mass Spectrom..

[52] Becker K.W., Elling F.J., Yoshinaga M.Y., Söllinger A., Urich T., Hinrichs K.-U. (2016). Unusual butane- and pentanetriol-based tetraether lipids in *Methanomassiliicoccus luminyensis*, a representative of the seventh order of methanogens. Appl. Environ. Microbiol..

[53] Coffinet S., Meador T.B., Mühlena L., Becker K.W., Schröder J., Zhu Q.-Z., Lipp J.S., Heuer V.B., Crump M.P., Hinrichs K.-U. (2020). Structural elucidation and environmental distributions of butanetriol and pentanetriol dialkyl glycerol tetraethers (BDGTs and PDGTs). Biogeosciences.

[54] Coffinet S., Mühlena L., Lipp J.S., Weil M., Neubauer C., Urich T., Hinrichs K.-U. (2022). Evidence for enzymatic backbone methylation of the main membrane lipids in the archaeon *Methanomassiliicoccus luminyensis*. Appl. Environ. Microbiol..

[55] Liu X.-L., Lipp J.S., Simpson J.H., Lin Y.-S., Summons R.E., Hinrichs K.-U. (2012). Mono-and dihydroxyl glycerol dibiphytanyl glycerol tetraethers in marine sediments: Identification of both core and intact polar lipid forms. Geochim. Cosmochim. Acta.

[56] Elling F.J., Könneke M., Nicol G.W., Stieglmeier M., Bayer B., Spieck E., de la Torre J.R., Becker K.W., Thomm M.J., Prosser J.I. (2017). Chemotaxonomic characterisation of the thaumarchaeal lipidome. Environ. Microbiol..

[57] Xiao W., Xu Y., Zhang C., Lin J., Wu W., Xiaoxia L., Tan J., Zhang X., Zheng F., Song X. (2023). Disentangling effects of sea surface temperature and water depth on hydroxylated isoprenoid GDGTs: Insights from the Hadal zone and global sediments. Geophys. Res. Lett..

[58] Koga Y., Nishihara M., Morii H., Akagawa-Matsushita M. (1993). Ether polar lipids of methanogenic bacteria: Structures, comparative aspects, and biosyntheses. Microbiol. Rev..

[59] Scholte A., Hübner C., Ströhl D., Scheufler O., Czich S., Börke J.M., Hildebrand G., Liefeith K. (2021). First isolation and structure elucidation of GDNT-β-Glu—Tetraether lipid fragment from archaeal *Sulfolobus* strains. Chem. Open.

[60] Řezanka T., Kolouchova I., Gharwalova L., Palyzova A., Sigler K. (2018). Lipidomic analysis: From archaea to mammals. Lipids.

[61] Law K.P., Zhang C.L. (2019). Current progress and future trends in mass spectrometry-based archaeal lipidomics. Org. Geochem..

[62] Lobasso S., Lopalco P., Angelini R., Vitale R., Huber H., Müller V., Corcelli A. (2012). Coupled TLC and MALDI-TOF/MS analyses of the lipid extract of the hyperthermophilic archaeon *Pyrococcus furiosus*. Archaea.

[63] Tourte M., Kuentz V., Schaeffer P., Grossi V., Cario A., Oger P.M. (2020). Novel intact polar and core lipid compositions in the *Pyrococcus* model species, *P. furiosus* and *P. yayanosii*, reveal the largest lipid diversity amongst Thermococcales. Biomolecules.

[64] Tourte M., Schaeffer P., Grossi V., Oger P.M. (2020). Functionalized membrane domains: An ancestral feature of Archaea?. Front. Microbiol..

[65] Cario A., Grossi V., Schaeffer P., Oger P.M. (2015). Membrane homeoviscous adaptation in the piezo-hyperthermophilic archaeon *Thermococcus barophilus*. Front. Microbiol..

[66] Bauersachs T., Weidenbach K., Schmitz R.A., Schwark L. (2015). Distribution of glycerol ether lipids in halophilic, methanogenic and hyperthermophilic archaea. Org. Geochem..

[67] Yao W., Zhang W., He W., Xiao W., Chen Y., Zhu Y., Zheng F., Zhang C. (2023). Lipidomic chemotaxonomy aligned with phylogeny of Halobacteria. Front. Microbiol..

[68] Naafs B.D.A., McCormick D., Inglis G.N., Pancost R.D. (2018). Archaeal and bacterial H-GDGTs are abundant in peat and their relative abundance is positively correlated with temperature. Geochim. Cosmochim. Acta.

[69] Chugunov A.O., Volynsky P.E., Krylov N.A., Boldyrev I.A., Efremov R.G. (2014). Liquid but durable: Molecular dynamics simulations explain the unique properties of archaeal-like membranes. Sci. Rep..

[70] Rastädter K., Wurm D.J., Spadiut O., Quehenberger J. (2020). The cell membrane of *Sulfolobus* spp. Homeoviscous adaption and biotechnological applications. Int. J. Mol. Sci..

[71] Kapoor B., Gupta R., Gulati M., Singh S.K., Khursheed R., Gupta M. (2019). The Why, Where, Who, How, and What of the vesicular delivery systems. Adv. Colloid Interface Sci..

[72] Jacquemet A., Barbeau J., Lemiègre L., Benvegnu T. (2009). Archaeal tetraether bipolar lipids: Structures, functions and applications. Biochimie.

[73] Kashyap K. (2021). Archaeosomes: Revolutionary technique for both cell-based and drug-based delivery applications. Int. J. Pharm. Sci. Med..

[74] Kaur G., Garg T., Rath G., Goyal A.K. (2016). Archaeosomes: An excellent carrier for drug and cell delivery. Drug Deliv..

[75] Santhosh P.B., Genova J. (2022). Archaeosomes: New generation of liposomes based on archaeal lipids for drug delivery and biomedical applications. ACS Omega.

[76] Chong P.L.-G., Chang A., Yu A., Mammedova A. (2022). Vesicular and planar membranes of archaea lipids: Unusual physical properties and biomedical applications. Int. J. Mol. Sci..

[77] Adamiak N., Krawczyk K.T., Locht C., Kowalewicz-Kulbat M. (2021). Archaeosomes and gas vesicles as tools for vaccine development. Front. Immunol..

[78] Ayesa U., Chong P.L.-G. (2020). Polar lipid fraction E from *Sulfolobus acidocaldarius* and dipalmitoylphosphatidylcholine can form stable yet thermo-sensitive tetraether/diester hybrid archaeosomes with controlled release capability. Int. J. Mol. Sci..

[79] Satyanarayana T., Raghukumar C., Shivaji S. (2005). Extremophilic microbes: Diversity and perspectives. Curr. Sci..

[80] Koga Y. (2012). Thermal adaptation of the archaeal and bacterial lipid membranes. Archaea.

[81] Miura Y. (2013). The biological significance of ω-oxidation of fatty acids. Proc. Jpn. Acad. Ser. B.

[82] Kaneda T. (1991). *Iso*-and *anteiso*-fatty acids in bacteria: Biosynthesis, function, and taxonomic significance. Microbiol. Rev..

[83] Clarke N.G., Hazlewood G.P., Dawson R.M.C. (1980). Structure of diabolic acid-containing phospholipids isolated from *Butyrivibrio* sp. Biochem. J..

[84] Sinninghe Damsté J.S., Rijpstra W.I.C., Hopmans E.C., Schouten S., Balk M., Stams A.J. (2007). Structural characterization of diabolic acid-based tetraester, tetraether and mixed ether/ester, membrane-spanning lipids of bacteria from the order Thermotogales. Arch. Microbiol..

[85] Sahonero-Canavesi D.X., Villanueva L., Bale N.J., Bosviel J., Koenen M., Hopmans E.C., Sinninghe Damsté J.S. (2022). Changes in the distribution of membrane lipids during growth of *Thermotoga maritima* at different temperatures: Indications for the potential mechanism of biosynthesis of ether-bound diabolic acid (membrane-spanning) lipids. Appl. Environ. Microbiol..

[86] Sinninghe Damsté J.S., Rijpstra W.I.C., Hopmans E.C., Weijers J.W., Foesel B.U., Overmann J., Dedysh S.N. (2011). 13,16-Dimethyl octacosanedioic acid (*iso*-diabolic acid), a common membrane-spanning lipid of Acidobacteria subdivisions 1 and 3. Appl. Environ. Microbiol..

[87] Weijers J.W., Schouten S., Hopmans E.C., Geenevasen J.A., David O.R., Coleman J.M., Pancost R.D., Sinninghe Damsté J.S. (2006). Membrane lipids of mesophilic anaerobic bacteria thriving in peats have typical archaeal traits. Environ. Microbiol..

[88] Weijers J.W., Panoto E., van Bleijswijk J., Schouten S., Rijpstra W.I.C., Balk M., Stams J.W.H., Rijpstra W.I., Sinninghe Damsté J.S. (2009). Constraints on the biological source(s) of the orphan branched tetraether membrane lipids. Geomicrobiol. J..

[89] De Jonge C., Hopmans E.C., Stadnitskaia A., Rijpstra W.I.C., Hofland R., Tegelaar E., Sinninghe Damsté J.S. (2013). Identification of novel penta-and hexamethylated branched glycerol dialkyl glycerol tetraethers in peat using HPLC–MS2, GC–MS and GC–SMB-MS. Org. Geochem..

[90] Peterse F., van der Meer J., Schouten S., Weijers J.W., Fierer N., Jackson R.B., Kim J.H., Sinninghe Damsté J.S. (2012). Revised calibration of the MBT–CBT paleotemperature proxy based on branched tetraether membrane lipids in surface soils. Geochim. Cosmochim. Acta.

[91] Zhao B., Russell J.M., Tsai V.C., Blaus A., Parish M.C., Liang J., Wilk A., Du X., Bush M.B. (2023). Evaluating global temperature calibrations for lacustrine branched GDGTs: Seasonal variability, paleoclimate implications, and future directions. Quat. Sci. Rev..

[92] Chen Y., Zheng F., Yang H., Yang W., Wu R., Liu X., Liang H., Chen H., Pei H., Zhang C. (2022). The production of diverse brGDGTs by an Acidobacterium providing a physiological basis for paleoclimate proxies. Geochim. Cosmochim. Acta.

[93] Sahonero-Canavesi D.X., Siliakus M.F., Abdala Asbun A., Koenen M., von Meijenfeldt F.B., Boeren S., Bale N.J., Engelman J.C., Fiege K., von Schijndel L.S. (2022). Disentangling the lipid divide: Identification of key enzymes for the biosynthesis of membrane-spanning and ether lipids in Bacteria. Sci. Adv..

[94] Peterse F., Hopmans E.C., Schouten S., Mets A., Rijpstra W.I.C., Sinninghe Damsté J.S. (2011). Identification and distribution of intact polar branched tetraether lipids in peat and soil. Org. Geochem..

[95] Makarieva T.N., Denisenko V.A., Stonik V.A., Milgrom Y.N., Rashkes Y.V. (1989). Rhizochalin, a novel secondary metabolite of mixed biosynthesis from the sponge *Rhizochalina incrustata*. Tetrahedron Lett..

[96] Makarieva T.N., Dmitrenok P.S., Zakharenko A.M., Denisenko V.A., Guzii A.G., Li R., Skepper C.R., Molinski T.F., Stonik V.A. (2007). Rhizochalins C and D from the sponge *Rhizochalina incrustata*. A rare *threo*-sphingolipid and a facile method for determination of the carbonyl position in α,ω-bifunctionalized ketosphingolipids. J. Nat. Prod..

[97] Harrison P.J., Dunn T.M., Campopiano D.J. (2018). Sphingolipid biosynthesis in man and microbes. Nat. Prod. Rep..

[98] Molinski T.F., Makarieva T.N., Stonik V.A. (2000). (−)-Rhizochalin is a dimeric enantiomorphic (2*R*)-sphingolipid: Absolute configuration of pseudo-C2v-symmetric bis-2-amino-3-alkanols by CD. Angew. Chem..

[99] Makarieva T.N., Zakharenko A.M., Dmitrenok P.S., Guzii A.G., Denisenko V.A., Savina A.S., Dalisay D.S., Molinski T.F., Stonik V.A. (2009). Isorhizochalin: A minor unprecedented bipolar sphingolipid of stereodivergent biogenesis from the *Rhizochalina incrustata*. Lipids.

[100] Makarieva T.N., Guzii A.G., Denisenko V.A., Dmitrenok P.S., Santalova E.A., Pokanevich E.V., Molinski T.F., Stonik V.A. (2005). Rhizochalin A, a novel two-headed sphingolipid from the sponge *Rhizochalina incrustata*. J. Nat. Prod..

[101] Makarieva T.N., Guzii A.G., Denisenko V.A., Dmitrenok P.S., Stonik V.A. (2008). New two-headed sphingolipid-like compounds from the marine sponge *Oceanapia* sp. Russ. Chem. Bull..

[102] Jin J.O., Shastina V., Park J.I., Han J.Y., Makarieva T., Fedorov S., Rasskazov V., Stonik V., Kwak J.Y. (2009). Differential induction of apoptosis of leukemic cells by rhizochalin, two headed sphingolipids from sponge and its derivatives. Biol. Pharm. Bull..

[103] Dyshlovoy S.A., Otte K., Tabakmakher K.M., Hauschild J., Makarieva T.N., Shubina L.K., Fedorov S.N., Bokemeyer C., Stonik V.A., von Amsberg G. (2018). Synthesis and anticancer activity of the derivatives of marine compound rhizochalin in castration resistant prostate cancer. Oncotarget.

[104] Khanal P., Kang B.S., Yun H.J., Cho H.G., Makarieva T.N., Choi H.S. (2011). Aglycon of rhizochalin from the *Rhizochalina incrustata* induces apoptosis via activation of AMP-activated protein kinase in HT-29 colon cancer cells. Biol. Pharm. Bull..

[105] Fedorov S.N., Makarieva T.N., Guzii A.G., Shubina L.K., Kwak J.Y., Stonik V.A. (2009). Marine two-headed sphingolipid-like compound rhizochalin inhibits EGF-induced transformation of JB6 P+ Cl 41 cells. Lipids.

[106] Sibirtsev J.T., Shastina V.V., Menzorova N.I., Makarieva T.N., Rasskazov V.A. (2011). Ca^2+^, Mg^2+^-dependent DNAase involvement in apoptotic effects in spermatozoa of sea urchin Strongylocentrotus intermedius induced by two-headed sphingolipid rhizochalin. Mar. Biotechnol..

[107] Makarieva T.N., Ivanchina N.V., Stonik V.A. (2020). Application of oxidative and reductive transformations in the structure determination of marine natural products. J. Nat. Prod..

[108] Nicholas G.M., Hong T.W., Molinski T.F., Lerch M.L., Cancilla M.T., Lebrilla C.B. (1999). Oceanapiside, an antifungal bis-α,ω-amino alcohol glycoside from the marine sponge *Oceanapia phillipensis*. J. Nat. Prod..

[109] Nicholas G.M., Molinski T.F. (2000). Enantiodivergent biosynthesis of the dimeric sphingolipid oceanapiside from the marine sponge *Oceanapia phillipensis*. Determination of remote stereochemistry. J. Am. Chem. Soc..

[110] Dalisay D.S., Rogers E.W., Molinski T.F. (2021). Oceanapiside, a marine natural product, targets the sphingolipid pathway of fluconazole-resistant *Candida glabrata*. Mar. Drugs.

[111] Zhou B.N., Mattern M.P., Johnson R.K., Kingston D.G. (2001). Structure and stereochemistry of a novel bioactive sphingolipid from a *Calyx* sp. Tetrahedron.

[112] Sugawara K., Watarai H., Ise Y., Yokose H., Morii Y., Yamawaki N., Matsunaga S. (2021). Structure elucidation of calyxoside B, a bipolar sphingolipid from a marine sponge *Cladocroce* sp. through the use of Beckmann rearrangement. Mar. Drugs.

[113] Kong F., Faulkner D.J. (1993). Leucettamols A and B, two antimicrobial lipids from the calcareous sponge *Leucetta microraphis*. J. Org. Chem..

[114] Dalisay D.S., Tsukamoto S., Molinski T.F. (2009). Absolute configuration of the α,ω-bifunctionalized sphingolipid leucettamol A from *Leucetta microrhaphis* by deconvoluted exciton coupled CD. J. Nat. Prod..

[115] Tsukamoto S., Takeuchi T., Rotinsulu H., Mangindaan R.E., van Soest R.W., Ukai K., Kobayashi H., Namikoshi M., Ohtsa T., Yokosawa H. (2008). Leucettamol A: A new inhibitor of Ubc13-Uev1A interaction isolated from a marine sponge, *Leucetta* aff. microrhaphis. Bioorg. Med. Chem. Lett..

[116] Nilius B., Owsianik G., Voets T., Peters J.A. (2007). Transient receptor potential cation channels in disease. Physiol. Rev..

[117] Chianese G., Fattorusso E., Putra M.Y., Calcinai B., Bavestrello G., Moriello A.S., De Petrocellis D., Di Marzo V., Taglialatela-Scafati O. (2012). Leucettamols, bifunctionalized marine sphingoids, act as modulators of TRPA1 and TRPM8 channels. Mar. Drugs.

[118] Makarieva T.N., Denisenko V.A., Dmitrenok P.S., Guzii A.G., Santalova E.A., Stonik V.A., MacMillan J.B., Molinski T.F. (2005). Oceanalin A, a hybrid α,ω-bifunctionalized sphingoid tetrahydroisoquinoline *β*-glycoside from the marine sponge *Oceanapia* sp. Org. Lett..

[119] Makarieva T.N., Ivanchina N.V., Dmitrenok P.S., Guzii A.G., Stonik V.A., Dalisay D.S., Molinski T.F. (2021). Oceanalin B, a hybrid α,ω-bifunctionalized sphingoid tetrahydroisoquinoline β-glycoside from the marine sponge *Oceanapia* sp. Mar. Drugs.

[120] Lievens S.C., Molinski T.F. (2005). Sagittamides A and B. Polyacetoxy long-chain acyl amino acids from a Didemnid Ascidian. Org. Lett..

[121] Lievens S.C., Molinski T.F. (2006). Progressive−convergent elucidation of stereochemistry in complex polyols. The absolute configuration of (−)-sagittamide A. J. Am. Chem. Soc..

[122] Seike H., Ghosht I., Kishi Y. (2006). Stereochemistry of sagittamide A: Prediction and confirmation. Org. Lett..

[123] Schuetz A., Junker J., Leonov A., Lange O.F., Molinski T.F., Griesinger C. (2007). Stereochemistry of sagittamide A from residual dipolar coupling enhanced NMR. J. Am. Chem. Soc..

[124] Lievens S.C., Morinaka B.I., Molinski T.F. (2010). Stereochemical elucidation of new sagittamides C–F from a Didemnid ascidian. Aust. J. Chem..

[125] Humbert A., Plé K., Harakat D., Martinez A., Haudrechy A. (2012). A further contribution to the study of sagittamide A: Synthesis of a pivotal intermediate belonging to a rare L-series. Molecules.

[126] Whitson E.L., Bugni T.S., Chockalingam P.S., Concepcion G.P., Feng X., Jin G., Herper M.K., Mangalindon G.C., McDonald L.A., Ireland C.M. (2009). Fibrosterol sulfates from the Philippine sponge *Lissodendoryx* (*Acanthodoryx*) *fibrosa*: Sterol dimers that inhibit PKCζ. J. Org. Chem..

[127] Ushiyama S., Umaoka H., Kato H., Suwa Y., Morioka H., Rotinsulu H., Losung F., Mangindaan R.E.P., De Foogd N.J., Yokosawa H. (2012). Manadosterols A and B, sulfonated sterol dimers inhibiting the Ubc13–Uev1A interaction, isolated from the marine sponge *Lissodendryx fibrosa*. J. Nat. Prod..

[128] Einarsdottir E., Liu H.-B., Freysdottir J., Gotfredsen C.H., Omarsdottir S. (2016). Immunomodulatory N-acyl dopamine glycosides from the icelandic marine sponge *Myxilla incrustans* collected at a hydrothermal vent site. Planta Med..

[129] Guzii A.G., Makarieva T.N., Denisenko V.A., Dmitrenok P.S., Popov R.S., Kuzmich A.S., Dyshlovoy S.A., von Amsberg G., Krasokhin V.B., Stonik V.A. (2016). Melonoside A: An ω-glycosylated fatty acid amide from the Far Eastern marine sponge *Melonanchora kobjakovae*. Org. Lett..

[130] Guzii A.G., Makarieva T.N., Denisenko V.A., Dmitrenok P.S., Popov R.S., Kuzmich A.S., Krasokhin V.B., Kim N.Y., Stonik V.A. (2018). Melonoside B and melonosins A and B, lipids containing multifunctionalized ω-hydroxy fatty acid amides from the far eastern marine sponge *Melonanchora kobjakovae*. J. Nat. Prod..

[131] Guzii A.G., Makarieva T.N., Fedorov S.N., Menshov A.S., Denisenko V.A., Popov R.S., Iarotsckaia V.V., Kim N.Y., Stonik V.A. (2022). Toporosides A and B, cyclopentenyl-containing ω-glycosylated fatty acid amides, and toporosides C and D from the Northwestern Pacific marine sponge *Stelodoryx toporoki*. J. Nat. Prod..

[132] Fuhrhop J.H., Wang T. (2004). Bolaamphiphiles. Chem. Rev..

[133] Nuraje N., Bai H., Su K. (2013). Bolaamphiphilic molecules: Assembly and applications. Prog. Polym. Sci..

[134] Dhasaiyan P., Prasad B.L.V. (2017). Self-Assembly of bolaamphiphilic molecules. Chem. Rec..

[135] Goergen N., Wojcik M., Drescher S., Pinnapireddy S.R., Brüßler J., Bakowsky U., Jedelská J. (2019). The use of artificial gel forming bolalipids as novel formulations in antimicrobial and antifungal therapy. Pharmaceutics.

